# A framework for the estimation of treatment costs of cardiovascular conditions in the presence of disease transition

**DOI:** 10.1007/s10479-022-04914-x

**Published:** 2022-08-23

**Authors:** Mohit Goswami, Yash Daultani, Sanjoy Kumar Paul, Saurabh Pratap

**Affiliations:** 1 Operations Management Group, Indian Institute of Management Raipur, Abhanpur, India; 2 Operations Management Group, Indian Institute of Management Lucknow, Lucknow, India; 3 UTS Business School, University of Technology Sydney, Sydney, Australia; 4 Department of Mechanical Engineering, Indian Institute of Technology (BHU), Varanasi, India

**Keywords:** Medical decision-making, Healthcare systems, Resource planning, Markovian analysis

## Abstract

The current research aims to aid policymakers and healthcare service providers in estimating expected long-term costs of medical treatment, particularly for chronic conditions characterized by disease transition. The study comprised two phases (qualitative and quantitative), in which we developed linear optimization-based mathematical frameworks to ascertain the expected long-term treatment cost per patient considering the integration of various related dimensions such as the progression of the medical condition, the accuracy of medical treatment, treatment decisions at respective severity levels of the medical condition, and randomized/deterministic policies. At the qualitative research stage, we conducted the data collection and validation of various cogent hypotheses acting as inputs to the prescriptive modeling stage. We relied on data collected from 115 different cardio-vascular clinicians to understand the nuances of disease transition and related medical dimensions. The framework developed was implemented in the context of a multi-specialty hospital chain headquartered in the capital city of a state in Eastern India, the results of which have led to some interesting insights. For instance, at the prescriptive modeling stage, though one of our contributions related to the development of a novel medical decision-making framework, we illustrated that the randomized versus deterministic policy seemed more cost-competitive. We also identified that the expected treatment cost was most sensitive to variations in steady-state probability at the “major” as opposed to the “severe” stage of a medical condition, even though the steady-state probability of the “severe” state was less than that of the “major” state.

## Introduction

According to the world health organization (WHO), the work on healthcare costing and accompanying efficiencies explores questions around the usage of healthcare resources, particularly in the public health sector (WHO, 2022a). Strategies and policies for improving health by expanding access to healthcare services need to be looked at from a resource-centric perspective to remain viable, efficient, and affordable. Addressing the resource allocation and proactive estimation of expenditures for delivering healthcare services revolves around treatment policies, costs, and an economic assessment of contributions made to improving health, particularly chronic diseases (e.g., HIV, diabetes, and cardiovascular diseases). In particular, questions like how much needs to be allocated to healthcare spending to reach healthcare goals, what the cost drivers are, and how resources should be apportioned are essential questions that need to be addressed. Answers to such questions can be addressed by developing methods, tools, and frameworks. These can be integrated at a larger policy level so that efficient cost estimation can percolate at various levels, such as the country, district, and hospital levels (WHO, 2022b).

Global healthcare spending has been spiraling since the early 2000s, as per a report released by the WHO in 2019 (WHO Working Paper, 2019). Of particular concern is that healthcare costs have been continuing to rise at a much higher rate than the rate of increase in countries’ gross domestic product (GDP), irrespective of whether the country is a low-, middle-, or high-income country. For instance, between 2000 and 2017, the average increase in GDP for low-income countries was slightly higher than 6%. In the same period, the average increase in healthcare spending for such countries was somewhat less than 8%. In the case of high-income countries, the gap between the average increase in healthcare spending and the average increase in GDP is even more pronounced. Between 2000 and 2017, the average increase in GDP for high-income countries was less than 2%, while the corresponding increase in healthcare expenditure was almost 4%. The rise in healthcare spending worldwide continues to have positive effects on public health outcomes, particularly in developing nations (Dhagarra et al., 2019). However, increases in spending do not necessarily imply enhanced service coverage (WHO Working Paper, 2019).

Though increased healthcare spending can positively impact (both at the private- and public-sector level), there are important accompanying downsides, both at the macro and micro levels. At the macro level, healthcare spending has been accompanied by a significant increase in catastrophic and out-of-pocket spending (OOPS) as a share of total health expenditure in the past decade (WHO Working Paper, 2019). Catastrophic spending measures a household’s financial hardship. It reflects the concerns of households in having to choose between spending on healthcare services/products and spending on basic needs such as food, education, and housing. OOPS pertains to payment by households to healthcare service providers to obtain service and health products in cash or credit payments to doctors, pharmacies, and user fees (Walker et al., 2019). Globally, the number of individuals with catastrophic health spending rose between 2000 and 2015 (WHO Working Paper, 2019). Further, an increase in healthcare expenditure, particularly governmental spending, does not yield desirable effects until the right priority in budgetary allocation is set (WHO Working Paper, 2019). For instance, in the United States (US), the sickest 5% of the population consumes 50% of total healthcare costs, while the healthiest 50% of the population consumes only 3% of the nation’s healthcare costs. With lifestyle changes across the globe, chronic diseases such as diabetes and heart disease have soared, thus leading to spiraling costs (Balta et al., 2021). In the US, it is estimated that healthcare costs accompanied by productivity losses will cost the US economy more than $1 trillion annually (Raghupathi & Raghupathi, 2018). At a micro level, planning for healthcare costs, including those related to post-treatment, could significantly influence the allocation of resources in both the short- and long-term (Li et al., 2021; Mitropoulos et al., 2020). Therefore considering the challenges associated with resource planning, some measure of broad estimates of costs within a certain time period for a particular type of medical condition (especially chronic conditions) would be of tremendous value in that such reasonably estimated expected costs per patient would ensure a significant reduction in uncertainty in planning when considering stakeholders such as individuals, hospitals, and insurance companies (Chapel et al., 2017; Kwon et al., 2016; Mitropoulos et al., 2020; Tortorella et al., 2021). The aforementioned arguments warrant that for chronic diseases, in particular, the modeling and prediction of costs due to disease accrued over time is critical for future services and budgets. However, it is also well documented that cost-data modeling is often problematic due to gaps in the unavailability and distribution of such data (Cooper et al., 2006). Particularly in the ongoing COVID-19 pandemic, wherein countries have been faced with multiple waves, each wave accompanied by huge escalations in public and private expenditures, such cost modeling and prediction would enable governmental and household entities to have some degree of certainty in earmarking their budgets.

Any typical chronic medical condition such as cardiovascular disease is often characterized by different severity states such as minor, moderate, or severe. Over a period of time, these states can either deteriorate considerably or stay the same with certain frequencies (probabilities). There would be a certain level of treatment decision in each of these states depending on the extent of medical, medicinal, and surgical interventions. In such transitions, the expected costs, therefore, likely depend on whether treatment decisions are deterministic or randomized in nature. A deterministic decision reflects when the healthcare service provider prescribes an appropriate level of treatment depending on past experiences in that a certain expected cost of medical treatment would result.

On the other hand, a randomized policy pertains to an attempt by the medical service provider so that certain acceptable treatment decisions can be mapped to individual states of the medical condition to minimize the expected treatment costs. Further, long-term steady-state probabilities (i.e., probabilities [frequencies] with which the medical condition exists in a particular condition), would significantly impact the expected costs of treatment under both deterministic and randomized policies. Furthermore, data related to the accuracy of diagnosis can also impact the long-term steady-state probabilities and, therefore, treatment costs (Šimundić, 2009).

The following key research questions were developed in view of the aforementioned arguments and motivations.

RQ1: How can we model deterministic and randomized medical treatment policies by integrating different states of a medical condition, transition probabilities, the accuracy of medical treatment, and the costs associated with medical treatment, thus evolving a structured approach to predict and model medical costs over a period of time?

RQ2: How can we deploy and validate data related to probabilistic transitions and frequencies pertaining to various states of a medical condition such that, based on robust statistical validation, these can be adopted to illustrate our prescriptive modeling approach?

**Fig. 1:**
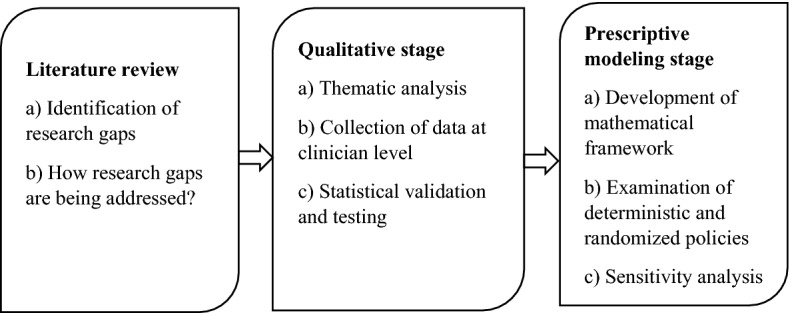
Progression of the study

To respond to these research questions, we specifically considered the case of patients accompanied by cardiovascular conditions who received treatment from one of the chains of a large multi-specialty hospital chain headquartered in the capital city of a state in Eastern India. In the first stage of our research (i.e., the qualitative stage), we employed the theoretical construct of thematic analysis to identify and classify major cardiovascular condition states. The clinicians for this classification were 115 retired cardiovascular specialists and surgeons (out of a total of 187 specialists who were contacted). Using the clinicians’ expert inputs, we also obtained a sense of the broad frequencies of each of these states, transitions, and accuracy of diagnoses. We further conducted statistical validations to eliminate outliers to ensure that the data from the remaining clinicians were not statistically different. Following this, in the second stage of our research (i.e., the modeling stage), using the linear programming (LP)-based exact-method technique, we modeled the frameworks of the minimization of expected medical costs of treatment under both deterministic and randomized policies. Using the data-related costs, transition probabilities, and so on, we illustrated the workings of the models developed. Figure 1 illustrates the progression of our study.

From Fig. 1, it can be seen our study involved three distinctly different phases. In the first phase, and based on the literature review, we identified the current research gaps and discussed how these gaps can be addressed. Following this, in the qualitative stage, based on semi-structured interviews with experts (cardiovascular clinicians), we conducted a thematic analysis focused on identifying major themes of the transition of the medical condition. We further collected the data pertaining to the prevalence of frequencies of medical conditions in various states, accompanying transitions, and so on. Finally, we performed statistical validation and testing of several hypotheses in the qualitative stage that formed the premise for the next study stage (i.e., the prescriptive modeling stage). We developed Markovian property-based mathematical models with consideration for both deterministic and randomized policies at the prescriptive modeling stage before performing a sensitivity analysis.

The remainder of the paper is organized as follows: Sect. 2 presents the literature review and research gaps; the research methodology is detailed in Sect. 3; Sect. 4 captures the step-wise solution methodology with an illustrative example; Sect. 5 enumerates the key findings and analysis; implications in terms of managerial and policy dimensions are discussed in Sect. 6; and, finally, the paper concludes in Sect. 7, wherein concluding remarks and future research directions are presented.

## Literature review and research gaps

The two primary research streams around which our research revolves are a) the cost and economic modeling of healthcare using analytical and statistical methods; b) the cost-dominant medical decision-making in healthcare. We now present the extant research literature related to both streams.

### Cost and economic modeling of healthcare using analytical and statistical methods

Using the Bayesian Markov-chain Monte Carlo simulation method, Cooper et al. (2007) devised a cost modeling method for diseases that are spread over time. The study contributed to the extant literature in two important ways from a methodological perspective. First, the regression-based statistical method developed was adjusted for modeling skewness (particularly a right-leaning skew) observed in the data. Second, the methods developed also accounted for the correlation of costs over different times for individuals. The model was used in the context of early inflammatory polyarthritis. From conducting a systematic review, Wammes et al. (2018) concluded that healthcare cost modeling does not only represent novel methodological outcomes. This is important from the standpoint that high-cost patients are typically sickest in that they represent higher utilization of scarce healthcare resources. Using data from high- as well as low-income countries, the study advocated for tailored interventions (as opposed to standard interventions) for high-cost patients. Lin et al. (2019) developed a machine-learning model to identify high-cost patients by incorporating expert knowledge about causal relationships. In particular, the study considered four types of variables with a high degree of linkage with the future high cost of treatment: procedure, demographic, diagnosis, and cost variables. An important contribution of this study was that it modeled cost implications for nonlinear and high-dimensional data using predictive modeling in the context of chronic obstructive pulmonary disease. Manrique-Rodriguez et al. (2014), using the severity levels and associated probabilities of adverse events, highlighted that cost savings during clinical judgment are often underestimated in current research in that such research does not typically consider indirect and intangible costs. Using various statistical methodologies, the study illustrated the cost-effectiveness of the implementation of smart-pump technology in pediatric intensive care units (ICUs). In their study on predicting healthcare costs, Revels et al. (2017) employed an autoregressive integrated moving average (ARIMA) time series analysis to model and forecast the future direct and indirect healthcare costs related specifically to morbid obesity. An important contribution of this study pertained to the identification of accelerating trends related to morbid obesity. Verma et al. (2022) described the descriptive and descriptive framework to analyze the demand for customer review ratings. Weaver et al. (2015) employed regression models to estimate the costs attributable to hypertension, adjusting for comorbidities and socio-demographic factors in the context of Canadian data up until 2020. The study demonstrated that hypertension accounts for significant spending in the Canadian healthcare budget and is projected to increase further as a percentage of the total budget. Employing a dataset from Barnes-Jewish Hospital, the study’s area under the curve (AUC) was found to be 0.70 for a 30-day readmission prediction. This AUC value was significantly higher than all extant line-based methods. Sushmita et al. (2015) employed machine learning algorithms to predict healthcare costs on publicly available survey data accurately. This study contributed to the extant literature in several ways. First, the study demonstrated that prior healthcare costs alone could be a good indicator of future healthcare costs. Second, the M5-model tree technique, as employed in the study, was found to generate accurate future healthcare cost predictions. Finally, the methods employed in the research were found to be useful in evaluating future costs for large segments of the population with reasonably low errors. Morid et al. (2018) identified five methods for predictive-cost modeling. Using a dataset of approximately 90,000 individuals and 6.3 million medical claims, the study demonstrated that gradient boosting had the best predictive performance for low- to medium-cost individuals. However, artificial neural networks (ANN) and ridge regression models worked better for high-cost individuals. Khalilpourazari et al. (2021) proposed a Gradient-based Grey Wolf Optimizer for complex optimization problems in that Gaussian walk and Levy flight were used to improve the exploration ability of evolved optimizers. The method developed was deployed in the context of COVID-19 case prediction in terms of the peak of infected, recovered, ICU-admitted, and death cases. They developed a Stochastic Fractal Search algorithm and combined it with a mathematical framework to forecast the pandemic based on public datasets to model the COVID-19 pandemic in Canada. The study showed that increasing testing capacity can enhance the detection of cases, particularly asymptomatic cases that mostly contribute to a rise in infections. They deployed a novel mathematical model to design an efficient flood evacuation plan in disasters. The problem was non-polynomial (NP) and hard in nature in that four different algorithms were offered to solve the mathematical model developed. The mathematical framework was also deployed to validate the real-life data from the devastating tsunami in Ishinomaki, Japan, in 2011.

### Cost-dominant medical decision-making in healthcare

In a study related to healthcare budgets and the decision rules of cost-effective healthcare providers, Baal et al. (2014) converged on the fact that most economic evaluations typically do not include all medical costs that may result in future costs related to illnesses. To this end, the study developed theoretical models and demonstrated optimal decision rules for cost-effectiveness analysis such that future costs of both related and unrelated medical care should be included. The theoretical model was applied to an example of transcatheter aortic valve implantation. Wang et al. (2018) employed cost-sensitive deep learning methods (grounded in convolution neural networks) that trained a multilayer perceptron (MLP) for readmission policy prediction. A key limitation that this study addressed was that, as opposed to a policy of relying on certain vital signs and diseases by extracting statistical features, it advocated for considering the skewness of class labels in medical data and the different costs of classification errors. Douglas (2020) showcased that responsibility-sensitive healthcare funding as a key lever for both patients and service providers goes a long way to ensure long-term healthcare costs remain at sustainable levels. Notably, this study also emphasized policy integration, particularly in chronic diseases, to enhance cost competitiveness and survival rates. Stadhouders et al. (2016) advocated for cost-containment policies for long-term cost control in the healthcare sector. In particular, the study emphasized four primary targets to contain costs, viz. volume controls, price controls, budgeting, and market-oriented policies. Daultani et al., (2015a, 2015b) introduced another dimension to reducing waiting times for patients based on simulation. Sari et al. (2017) analyzed lean-based policies and accounted for direct costs such as fees paid to consultants/other relevant expenses and indirect costs related to wages. Their work was further extended by Henrique et al. (2021), who had a view of specifically carrying out continuous improvement in healthcare, thereby contributing to the notion of lean healthcare with the objectives of minimizing both service provider and patient expenditure. Daultani et al., (2015a, 2015b) presented a comprehensive study on the detailed scope of lean applications in different healthcare settings. Cookson et al. (2017) deployed cost-effectiveness analysis (CEA) to address health equity concerns. The premise of this study revolved around ensuring social equity policies. It described two main ways to address health equity concerns using CEA. The first way pertains to equity impact analysis, which quantifies the distribution of cause and effect by equity-relevant variables. The second pertains to equity trade-off analysis, which quantifies trade-offs between improving total health and other equity objectives.

### Key research gaps and contributions

This section presents the research gaps addressed in the current study. Though studies such as Beaulieu and Bentahar (2021), Lin et al. (2019), and Walker et al. (2019) argue for robust modeling of economic costs and decision policies in healthcare service delivery, thereby understanding the economic impacts that can be of significant value both at the policy as well as operational level, we specifically expand on the extant literature in the following ways.

- Most current studies, including those by Lin et al. (2019), Morid et al. (2018), and Walker et al. (2019), implicitly assume that the policies around patient diagnosis and treatment are known well in advance, even for diseases that are subject to transition to degenerate states. This assumption around treatment policies given apriori is restrictive in that there cannot be scenario-based cost modeling of medical treatment. To address this limitation, the current study conducted cost modeling based on two kinds of treatment policies, viz. deterministic and randomized. Deterministic policies are those in which the healthcare professional performs treatment based on prescribed treatment guidelines. In contrast, in the randomized policy, treatment decisions can be made to keep direct expected costs at a minimum while still deploying acceptable treatment decisions for a particular medical condition state.
- Although de Gues et al. (2016) deployed Markovian decision analysis to contrast treatment strategies, the vast majority of extant studies that focus on cost and economic modeling failed to take into account the fact that chronic diseases can be associated with certain progression in that a specific medical condition can deteriorate to degenerate levels or remain at the same state. Further, information related to medical diagnosis serves as a key input in ascertaining the steady-state transition probabilities that in turn serve as a key input for the prescriptive modeling of expected treatment costs. Such harnessing of Markovian properties and assessment of deterministic and randomized policies, thus resulting in long-term steady-state probabilities, can be valuable to concerned stakeholders in that they can aid these stakeholders in understanding the economic impact of a medical condition (even at the localized level).
- Prior to developing the prescriptive framework based on linear optimization models, we collected real-life and empirically validated data on cardiovascular conditions from retired medical professionals (experts) who had experience diagnosing and treating such conditions. In particular, data on states of the said medical condition, transitions, and treatment accuracy were systematically collected during primary data collection. Further, we relied on robust statistical validation, thereby eliminating outliers and attempting to ensure that the data did not remain statistically dissimilar. All these nuances related to data and hypothesis formalized at the qualitative stage (stage 1) were key to establishing and validating the prescriptive model developed in stage 2. Therefore, an important contribution associated with our study is that, instead of evolving a prescriptive model characterized only by theoretical underpinnings, our model is grounded in real-life data backed further by strong statistical validation. Table 1 encapsulates a comparison of our research with the current literature.
- Most current studies, including those by Cooper et al. (2007), Lin et al. (2019), Sari et al. (2017), and Wang et al. (2018), provided a point-solution to optimal steady-state probabilities with consideration for various states of progression of the disease and did not perform post-optimality analyses. However, we also conducted a detailed sensitivity analysis to understand better the impact of variations in steady-state probabilities corresponding to different states. We further identified that the expected cost of treatment is most/least sensitive to a given state of the medical condition. Finally, in contrast to most current studies, by incorporating information related to true/false treatment, we also ensured that such dimensions were adequately captured at both the qualitative and prescriptive modeling stage.

**Table 1:** Comparison of our research with the current literature

Authors	Research context	Scope of study		Key research nuances			Methodical contributions	
		Sample/population -level study	Individual-level study	Disease transition	Accuracy of diagnosis	Treatment policies	Scope	Specific method deployed
Douglas et al. (2020)	Responsibility sensitive healthcare funding in long-term	✓				Responsibility by patients	Theoretical model	
Walker et al. (2019)	OOPS spending by households	✓					Qualitative study	Cross tabulation
Lin et al. (2019)	Prediction of high-cost patients using causal relationships		✓					
Wammes et al. (2018)	Modeling of costs for high-cost patients	✓					Qualitative study	
Morid et al. (2018)	Prediction of healthcare costs using supervised learning	✓			✓	Depending upon low-cost/high-cost patients	Predictive study	Machine learning algorithms, including ANN
Wang et al. (2018)	Cost-sensitivity methods for cost policies	✓			✓	Readmission Policy	Quantitative study	Multilayer perceptron
Sari et al. (2017)	Economic modeling of direct and indirect costs in healthcare		✓			Lean-based waste minimization policies	Theoretical model	
Stadhouders et al. (2016))	Cost containment policies for long-term cost control		✓			Volume, price, budget, and market-oriented policies		
Weaver et al. (2015)	Modeling of healthcare costs attributable to hypertension	✓					Predictive study	Regression-based model
Sushmita et al. (2015)	Prediction of healthcare costs using survey data	✓			✓		Predictive study	Machine learning
Baal et al. (2014)	Healthcare budgets and decision rules for effective healthcare services		✓			Based on related as well future costs	Theoretical model	
Cooper et al. (2007)	Predicting costs over time for early inflammatory polyarthritis	✓					Analytical & Statistical	Bayesian-Markovian method
Our study	Modeling healthcare costs using a two-stage mixed method	✓		✓	✓	Deterministic and randomized policies	Analytical and statistical	Linear optimization-based modeling based on statistical validation

✓ means a particular research dimension was captured in the study

## Research methodology

**Fig. 2:**
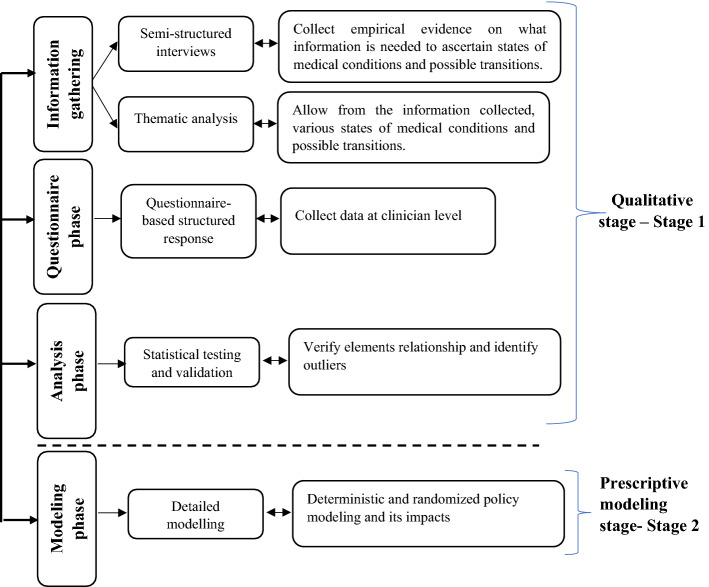
Schema of the research methodology

Practicality dictates that the most important determinants of research methodology are often formulated research questions (Saunders et al., 2019; Zhao et al., 2020). The research questions posed in the current study required the authors to move sequentially from empirical findings to mathematical modeling, thus forming an appropriate basis for research approaches intervening in the world and not merely observing the world (Goldkuhl, 2012; Zhao et al., 2020). Qualitative approaches have proven to be effective in gaining deep insights and diversified views of certain phenomena by probing experts (i.e., clinicians [in this case, a specific genre of medical professionals]) for their diversified views and understanding (Wellington & Szczerbinski, 2007). In line with the above assertions, we conducted the study in the qualitative stage (stage 1) and the modeling stage (stage 2), as shown in Fig. 2.

### Qualitative stage

The first stage, the qualitative stage, was aimed at data collection, validation, and preprocessing, thus serving as input to the second stage, the prescriptive modeling stage. In the first stage, one-on-one semi-structured interviews were conducted with experts (clinicians) over video calls (primarily due to the ongoing COVID-19 pandemic). The objective of such interviews as a primary method of data collection is anchored in the fact that such a method enables (1) insightful discussions with experienced medical professionals, thus obtaining richness in primary first-hand data; (2) the uncovering of newer knowledge through allowing clinicians to express their ideas freely; (3) the privacy of clinicians who are not willing to share personal experiences in front of peers; and (4) two-way communication between interviewer and interviewee. Following this, the use of thematic analysis helped identify, analyze, and report themes within the data. Further, thematic analysis has shown itself to be flexible and tangible, particularly in the context of qualitative data (Braun & Clarke, 2006).

At the end of the information-gathering phase, it was broadly expected that the major states of the medical condition along with its accompanying transitions would be finalized. These thematic inputs then acted as an input to collect the pertinent objective data, followed by rigorous statistical testing and validation to ensure that elemental relationships were identified and verified. In particular, we aimed to substantiate a few important questions: (a) *Are there any statistical differences in the frequencies of different states of medical conditions?*; (b) *Are there any statistical differences in the transitions of different states of medical conditions?*; and (c) *How can we work with the means of different parameters that are statistically similar by filtering out the outliers?*

Once the rigorous statistical validation was conducted, we moved to the second stage of the work (i.e., the prescriptive modeling stage), wherein we conducted detailed modeling of deterministic and randomized policies, as detailed in Sect. 3.2.

### Modeling stage

#### Expressing states of the medical condition and transitions

The indices, parameters, and decision variables of the model are presented in Table 2. There are three major indices pertaining to the actual states of the medical condition, treatment corresponding to a particular state, and corresponding medical decision. The decision variable pertains to optimal steady-state probabilities and optimal steady-state transition probabilities under both deterministic and randomized policies.

**Table 2:** Indices, parameters, and decision variable set

*Indices*	
*A(m*_*t*_*)*	Actual state “*m*_*t*_” represents a particular state of the medical condition
T(*m*_*t*_)	Corresponding treatment for particular state “m_*t*_” of the medical condition
*k*	Medical decision “*k*” such that *k* = *1, 2, 3….K*
*Parameters*	
$[p\{ A(m_{t} ) \to A(m_{T} )]$	Mean transition probability of the medical condition from state “*m*_*t*_” to state “*m*_*T*_”
$p\{ A(m_{t} )\}$	Mean probability of medical condition remaining in state “*m*_*t*_” itself
*D(k, m*_*t*_*)*	Decision “*k*” exercised for the state of medical condition *“m*_*t*_*”* under the deterministic policy
*y(k, m*_*t*_*)*	Decision “*k*” exercised for the state of medical condition *“m*_*t*_*”* under the randomized policy such that *y(k, m*_*t*_*) ϵ (0,1)*
*C{A(m*_*t*_*)}*	The average cost of treatment corresponding to the actual state of medical condition *“m*_*t*_*”*
*E*^*Det*^*(C)*	The expected cost of the treatment considering the deterministic policy
*E*^*Rand*^*(C)*	The expected cost of the treatment considering the randomized policy
*Decision variable set*	
$\theta^{*} \{ A(m_{t} ) \to A(m_{T} )]$	Optimal steady-state transition probabilities from state “*m*_*t*_” to “*m*_*T*_” under the deterministic policy
$y^{*} \{ A(m_{t} ) \to A(m_{T} )]$	Optimal steady-state unconditional transition probabilities from state “*m*_*t*_” to “*m*_*T*_” under the randomized policy
$\pi^{*} \{ A(m_{t} )\}$	Optimal probabilities of state “*m*_*t*_” to remain in the same state under the deterministic policy
$y^{*} \{ A(m_{t} )\}$	Optimal probabilities of state “*m*_*t*_” to remain in the same state under the randomized policy

Suppose a patient suffering from a specific medical condition, state *A(m*_*t*_*)* requires the corresponding treatment. The treatment would be appropriate only if the corresponding treatment is *T(m*_*t*_*)*. This means that for the specific state *A(m*_*t*_*)*, only *T(m*_*t*_*)* would be appropriate, and no other treatment decisions such as *T(m*_*1*_*)*, *T(m*_*2*_*)*, … *T(m*_*t-1*_*)*, …, *T(m*_*t*+*1*_*)*,…. *T(m*_*T*_*)* would be acceptable. If *A(m*_*t*_*)* is mapped to *T(m*_*t*_*)*, then the treatment decision would be termed ‘true treatment.’ Otherwise, the treatment would be termed ‘false treatment.’ Table 3(a) depicts this mapping for various states of a medical condition and corresponding treatment decisions in that it represents the treatment probability matrix represented as $[p\{ A(m_{t} ),T(m_{t} )]$. An element of this matrix depicts whether the decision is true or false, along with the mean probability of a specific state of a medical condition and accompanying treatment. For instance, referring to Table 3(a), for the element lying at the intersection of *A(m*_*t*_*)* and *T(m*_*t*_*)*, the treatment is true, and the corresponding probability of treatment corresponding to medical condition *A(m*_*t*_*)* is *p{ A(m*_*t*_*)*, *T(m*_*t*_*)}.*

**Table 3:** Medical condition probabilities and transitions

(a) Medical condition and treatment probability matrix							
State of medical condition *A(m*_*t*_*)* $\downarrow$		Treatment as *T(m*_*t*_*)* $\to$					
		*T(m*_*1*_*)*	*T(m*_*2*_*)*	*T(.)*	*T(m*_*t*_*)*	*T(..)*	*T(m*_*T*_*)*
	*A(m*_*1*_*)*	True Treatment *p{A(m*_*1*_*),T(m*_*1*_*)}*	False treatment *p{A(m*_*1*_*),T(m2)}*	False treatment *p{A(m*_*1*_*),T(m*_*3*_*)}*	False treatment *p{A(m*_*1*_*),T(m*_*t*_*)}*	False treatment *p{A(m*_*1*_*),T(..)}*	False treatment *p{A(m*_*1*_*),T(m*_*T*_*)}*
	*A(m*_*2*_*)*	False treatment *p{A(m*_*2*_*),T(m*_*1*_*)}*	True treatment *p{A(m*_*2*_*),T(m2)}*	False treatment *p{A(m*_*2*_*),T(m*_*3*_*)}*	False treatment *p{A(m*_*2*_*),T(m*_*t*_*)}*	False treatment *p{A(m*_*2*_*),T(..)}*	False treatment *p{A(m*_*2*_*),T(m*_*T*_*)}*
	*A(.)*	False treatment *p{A(.),T(m*_*1*_*)}*	False treatment *p{A(.),T(m2)}*	True treatment *p{A(.),T(m*_*3*_*)}*	False treatment *p{A(.),T(m*_*t*_*)}*	False treatment *p{A(.),T(..)}*	False treatment *p{A(.),T(m*_*T*_*)}*
	*A(m*_*t*_*)*	False treatment *p{A(m*_*t*_*),T(m*_*1*_*)}*	False treatment *p{A(m*_*t*_*),T(m2)}*	False treatment *p{A(m*_*t*_*),T(m*_*3*_*)}*	True treatment *p{A(m*_*t*_*),T(m*_*t*_*)}*	False treatment *p{A(m*_*t*_*),T(..)}*	False treatment *p{A(m*_*t*_*),T(m*_*T*_*)}*
	*A(..)*	False treatment *p{A(..),T(m*_*1*_*)}*	False treatment *p{A(..),T(m2)}*	False treatment *p{A(..),T(m*_*3*_*)}*	False treatment *p{A(..),T(m*_*t*_*)}*	True treatment *p{A(..),T(..)}*	False treatment *p{A(..),T(m*_*T*_*)}*
	*A(m*_*T*_*)*	False Treatment *p{A(m*_*T*_*),T(m*_*1*_*)}*	False treatment *p{A(m*_*T*_*),T(m2)}*	False treatment *p{A(m*_*T*_*),T(m*_*3*_*)}*	False treatment *p{A(m*_*T*_*),T(m*_*t*_*)}*	False treatment *p{A(m*_*T*_*),T(..)}*	True treatment *p{A(m*_*T*_*),T(m*_*T*_*)}*

(b) Medical condition transition matrix						
State	*A(m*_*1*_*)*	*A(m*_*2*_*)*	*A(.)*	*A(m*_*t*_*)*	*A(..)*	*A(m*_*T*_*)*
*A(m*_*1*_*)*	$p\{ A(m_{1} )\}$	$p\{ A(m_{1} ) \to A(m_{2} )\}$	$p\{ A(m_{1} ) \to A(.)\}$	$p\{ A(m_{1} ) \to A(m_{t} )\}$	$p\{ A(m_{1} ) \to A(..)\}$	$p\{ A(m_{1} ) \to A(m_{T} )\}$
*A(m*_*2*_*)*	0	$p\{ A(m_{2} )\}$	$p\{ A(m_{2} ) \to A(.)\}$	$p\{ A(m_{2} ) \to A(m_{t} )\}$	$p\{ A(m_{2} ) \to A(..)\}$	$p\{ A(m_{2} ) \to A(m_{T} )\}$
*A(.)*	0	0	$p\{ A(.)\}$	$p\{ A(.) \to A(m_{t} )\}$	$p\{ A(.) \to A(..)\}$	$p\{ A(.) \to A(m_{T} )\}$
*A(m*_*t*_*)*	0	0	0	$p\{ A(m_{t} )\}$	$p\{ A(m_{t} ) \to A(..)\}$	$p\{ A(m_{t} ) \to A(m_{T} )\}$
*A(..)*	0	0	0	0	$p\{ A(..)\}$	$p\{ A(..) \to A(m_{T} )\}$
*A(m*_*T*_*)*	0	0	0	0	0	$p\{ A(m_{T} )\}$

(c) Treatment decision and state of medical condition mapping (under the deterministic policy)						
State	*D(1)*	*D(2)*	*D(.)*	*D(k)*	*D(*_*..*_*)*	*D(K)*
*A(m*_*1*_*)*	*D(1,m*_*1*_*)*	*D(2,m*_*1*_*)*	*D(.,m*_*1*_*)*	*D(k,m*_*1*_*)*	*D(..,m*_*1*_*)*	*D(K,m*_*1*_*)*
*A(m*_*2*_*)*	*D(1,m*_*2*_*)*	*D(2,m*_*2*_*)*	*D(.,m*_*2*_*)*	*D(k,m*_*2*_*)*	*D(..,m*_*2*_*)*	*D(K,m*_*2*_*)*
*A(.)*	*D(1,.)*	*D(2,.)*	*D(.,.)*	*D(k,.)*	*D(..,.)*	*D(K,.)*
*A(m*_*t*_*)*	*D(1,m*_*t*_*)*	*D(2,m*_*t*_*)*	*D(.,m*_*t*_*)*	*D(k,m*_*t*_*)*	*D(..,m*_*t*_*)*	*D(K,m*_*t*_*)*
*A(..)*	*D(1,..)*	*D(2,..)*	*D(.,..)*	*D(k,..)*	*D(..,..)*	*D(K,..)*
*A(m*_*T*_*)*	*D(1,m*_*T*_*)*	*D(2,m*_*T*_*)*	*D(.,m*_*T*_*)*	*D(k,m*_*T*_*)*	*D(..,m*_*T*_*)*	*D(K,m*_*T*_*)*

(d) Treatment decision and state of medical condition mapping (under deterministic policy)						
State	*D(1)*	*D(2)*	*D(.)*	*D(k)*	*D(*_*..*_*)*	*D(K)*
*A(m*_*1*_*)*	*y(1,m*_*1*_*)*	*y(2,m*_*1*_*)*	*y(.,m*_*1*_*)*	*y(k,m*_*1*_*)*	*y(..,m*_*1*_*)*	*y(K,m*_*1*_*)*
*A(m*_*2*_*)*	*y(1,m*_*2*_*)*	*y(2,m*_*2*_*)*	*y(.,m*_*2*_*)*	*y(k,m*_*2*_*)*	*y(..,m*_*2*_*)*	*y(K,m*_*2*_*)*
*A(.)*	*y(1,.)*	*y(2,.)*	*y(.,.)*	*y(k,.)*	*y(..,.)*	*y(K,.)*
*A(m*_*t*_*)*	*y(1,m*_*t*_*)*	*y(2,m*_*t*_*)*	*y(.,m*_*t*_*)*	*y(k,m*_*t*_*)*	*y(..,m*_*t*_*)*	*y(K,m*_*t*_*)*
*A(..)*	*y(1,..)*	*y(2,..)*	*y(.,..)*	*y(k,..)*	*y(..,..)*	*y(K,..)*
*A(m*_*T*_*)*	*y(1,m*_*T*_*)*	*y(2,m*_*T*_*)*	*y(.,m*_*T*_*)*	*y(k,m*_*T*_*)*	*y(..,m*_*T*_*)*	*y(K,m*_*T*_*)*

Another dimension of medical treatment is related to the Markovian property in that the state of a specific medical condition can evolve to other severe states of the specific medical condition. For example, an individual with a specific “minor” cardiovascular condition can remain in this same condition with a probability of 1/2 or can deteriorate to a “major” and “severe” conditions over a period of time with probabilities of 3/8 or 1/8, respectively. This means that, on average, there is a 50% likelihood of the patient remaining steady at the same “minor” condition. The patient’s “minor” condition would deteriorate to “major” and “severe” with a 37.5% and 12.5% likelihood, respectively. Referring to Table 3(b) and the transition matrix within $[p\{ A(m_{t} ) \to A(m_{T} )]$, *A(m*_*1*_*)* denotes a “minor condition”. *A(m*_*1*_*)* can remain at this state itself with a probability of *p{A(m*_*1*_*)}. A(m*_*1*_*)* can further deteriorate to lower states of the medical condition with individual probabilities. For instance, state *A(m*_*1*_*)* can deteriorate to a lower state *A(m*_*2*_*)* with a mean transition probability of $p\{ A(m_{1} ) \to A(m_{2} )\}$.

If *A(m*_*1*_*)*, *A(m*_*2*_*)*, *A(.)*, *A(m*_*t*_*)*, *A(m*_*T*_*)*, *A(..)*, *A(m*_*T*_) are all possible exhaustive states of a medical condition such that *A(m*_*1*_*)* >  > *A(m*_*2*_*)* >  > *A(.)* >  > *A(m*_*t*_*)* >  > *A(m*_*T*_*)* >  > *A(..)* >  > *A(m*_*T*_) with >  > depicting progressively degenerate states, then the sum of the corresponding mean transition probabilities corresponding to all the rows would be equal to 1. For instance, within the medical condition transition matrix $[p\{ A(m_{t} ) \to A(m_{T} )]$, for the first row in Table 3(b), the sum of the transition probabilities can be expressed using the following mathematical expression:

1 $$ \begin{aligned} & p\{ A(m_{1} )\} + p\{ A(m_{1} ) \to A(m_{2} )\} + p\{ A(m_{1} ) \to A(.)\} + p\{ A(m_{1} ) \to A(m_{t} )\} \\ &  \quad + p\{ A(m_{1} ) \to A(..)\} + p\{ A(m_{1} ) \to A(m_{T} )\} = 1 \\ \end{aligned} $$

Further, a particular degenerate state, for instance, *A(m*_*2*_*)* cannot transition back to better prior states. Therefore, the values of such transition probabilities would be equal to zero.

Similarly, for the remainder of the row, the following mathematical expressions can be used:

2 $$ \begin{aligned} & p\{ A(m_{2} )\} + p\{ A(m_{2} ) \to A(.)\} + p\{ A(m_{2} ) \to A(m_{t} )\} \\ & \quad + p\{ A(m_{2} ) \to A(..)\} + p\{ A(m_{2} ) \to A(m_{T} )\} = 1 \\ \end{aligned} $$

3 $$ \begin{aligned} & p\{ A(.)\} + p\{ A(.) \to A(m_{t} )\} + p\{ A(.) \to A(..)\} \\ & \quad + p\{ A(.) \to A(m_{T} )\} = 1 \\ \end{aligned} $$

4 $$ p\{ A(m_{t} )\} + p\{ A(m_{t} ) \to A(..)\} + p\{ A(m_{t} ) \to A(m_{T} )\} = 1 $$

5 $$ p\{ A(..)\} + p\{ A(..) \to A(m_{T} )\} = 1 $$

Equations (1)–(5) ensure that the sum of the transition probabilities corresponding to each state of the medical condition equals 1.

Notably, the last state, *A(m*_*T*_*)*, will not improve from any previous state and will remain in this state only with a probability of 1. Therefore, the value of *p{A(m*_*T*_*)}* would be equal to 1. The interplay of likelihoods of states of a medical condition and corresponding medical treatments and transition probabilities would have a combined effect in that the resulting probabilities would be indicative of uncertainties associated with a specific state of medical condition/corresponding treatment while at the same time taking into account deterioration to a degenerate state of a medical condition. This means that the effective transition probability would be a key input in subsequent modeling.

When we consider the mean effective transition probability, we can ascertain the elements of the effective transition probability using the independent property of the two probability element sets depicted in Table 3(a) and (b).

The effective transition probability can be represented using two types. The first is a transition within the same state (e.g., “minor” to “minor”), and the second is a transition from one state to another (e.g., state “minor” to “major”). The effective transition probability in the same state of a medical condition—for instance, related to state *A(m*_*1*_*)*—is expressed as *x{A(m*_*1*_*)}* and can be determined using the following mathematical expression:

6 $$ x\{ A(m_{1} )\} = \frac{{p\{ A(m_{1} ) \times p\{ A(m_{1} ),T(m_{1} )\} }}{\begin{gathered} p\{ A(m_{1} ) \times p\{ A(m_{1} ),T(m_{1} )\} + p\{ A(m_{1} ),T(m_{2} )\} \times p\{ A(m_{1} ) \to T(m_{2} )\} \hfill \\ + \cdots p\{ A(m_{1} ),T(m_{T} )\} \times p\{ A(m_{1} ) \to T(m_{T} )\} \hfill \\ \end{gathered} } $$

The effective transition probability when the state of a medical condition transitions to some other state, for instance, when state *A(m*_*1*_*)* transitions to *A(m*_*T*_*)*, is expressed as $x\{ A(m_{1} ) \to A(m_{T} )\}$ and can be determined using the following mathematical expression:

7 $$ x\{ A(m_{1} ) \to A(m_{T} )\} = \frac{{p\{ A(m_{1} ),T(m_{T} )\} \times p\{ A(m_{1} ) \to A(m_{T} )\} }}{\begin{gathered} p\{ A(m_{1} ) \times p\{ A(m_{1} ),T(m_{1} )\} + p\{ A(m_{1} ),T(m_{2} )\} \times p\{ A(m_{1} ) \to A(m_{2} )\} \hfill \\ + \cdots p\{ A(m_{1} ),T(m_{T} )\} \times p\{ A(m_{1} ) \to A(m_{T} )\} \hfill \\ \end{gathered} } $$

Similar to the approach specified in Eq. (7), the remaining elements of the effective medical condition transition matrix can be ascertained such that the matrix can be denoted as $[x\{ A(m_{t} ) \to A(m_{T} )]$. In tabular form, the matrix $[x\{ A(m_{t} ) \to A(m_{T} )]$ would look similar to Table 3(b), except *p* would be replaced by *x*.

#### Deterministic and randomized policies

##### Deterministic policies

Once the elements of the matrix $[x\{ A(m_{t} ) \to A(m_{T} )]$ are established, we can proceed to determine steady-state probabilities corresponding to individual states of a medical condition. These steady-state probabilities denote the long-term likelihood of medical conditions in a particular state. It is to be noted that *A(m*_*T*_*)* represents the absorbing state in that once the state of medical condition finally transitions to *A(m*_*T*_*)*, it becomes an infeasible state and needs to be brought back to state *A(m*_*1*_*).* For instance, if the patient goes into a “severe” state in certain cardiovascular conditions, the patient might have to be administered a pacemaker. Therefore, the corresponding probability for *A(m*_*1*_*)* would assume a value of 1.

If $\theta^{*} \{ A(m_{1} )\}$, $\theta^{*} \{ A(m_{2} )\}$, $\theta^{*} \{ A(.)\}$, $\theta^{*} \{ A(m_{t} )\}$, $\theta^{*} \{ A(..)\}$, and $\theta^{*} \{ A(m_{T} )\}$ are the mean steady-state probabilities corresponding to states $A(m_{1} )$, $A(m_{2} )$, $A(.)$, $A(m_{t} )$, $A(..)$, and $A(m_{T} )$ respectively, then the following mathematical expressions can be written to ascertain steady-state probabilities in line with Markovian theory (Hillier et al., 2010). These steady-state probabilities signify the likelihood of observing a particular state of a medical condition long-term:

8 $$ \theta^{*} \{ A(m_{1} )\} = \theta^{*} \{ A(m_{T} )\} $$

9 $$ \theta^{*} \{ A(m_{2} )\} = \theta^{*} \{ A(m_{1} )\} \times x\{ A(m_{1} ) \to A(m_{2} )\} + \theta^{*} \{ A(m_{2} )\} \times x\{ A(m_{2} )\} $$

10 $$ \begin{aligned} \theta^{*} \{ A(.)\} & = \theta^{*} \{ A(m_{1} )\} \times x\{ A(m_{1} ) \to A(.)\} + \theta^{*} \{ A(m_{2} )\} \times x\{ A(m_{2} ) \to A(.)\} \\ & \quad { + }\theta^{*} \{ A(.)\} \times x\{ A(.)\} \\ \end{aligned} $$

11 $$ \begin{aligned} \theta^{*} \{ A(m_{t} )\} & = \theta^{*} \{ A(m_{1} )\} \times x\{ A(m_{1} ) \to A(m_{t} )\} + \theta^{*} \{ A(m_{2} )\} \times x\{ A(m_{2} ) \to A(m_{t} )\} \\ & \quad { + }\theta^{*} \{ A(.)\} \times x\{ A(.) \to A(m_{t} )\} + \theta^{*} \{ A(m_{t} )\} \times x\{ A(m_{t} )\} \\ \end{aligned} $$

12 $$ \begin{aligned} \theta^{*} \{ A(..)\} & = \theta^{*} \{ A(m_{1} )\} \times x\{ A(m_{1} ) \to A(..)\} + \vartheta^{*} \{ A(m_{2} )\} \times x\{ A(m_{2} ) \to A(..)\} \\ & \quad { + }\theta^{*} \{ A(.)\} \times x\{ A(.) \to A(..)\} + \theta^{*} \{ A(m_{t} )\} \times x\{ A(m_{t} ) \to A(..)\} \\ & \quad { + }\theta^{*} \{ A(.)\} \times x\{ A(..)\} \\ \end{aligned} $$

13 $$ \begin{aligned} \theta^{*} \{ A(m_{T} )\} & = \theta^{*} \{ A(m_{1} )\} \times x\{ A(m_{1} ) \to A(m_{T} )\} + \theta^{*} \{ A(m_{2} )\} \times x\{ A(m_{2} ) \to A(m_{T} )\} \\ & \quad { + }\theta^{*} \{ A(.)\} \times x\{ A(.) \to A(m_{T} )\} + \theta^{*} \{ A(m_{t} )\} \times x\{ A(m_{t} ) \to A(m_{T} )\} \\ & \quad { + }\theta^{*} \{ A(..)\} \times x\{ A(..) \to A(m_{T} )\} \\ \end{aligned} $$

Equations (8)–(13) ensure that each of the steady-state probabilities is written as a sum of the probabilities of all the possible ways one state can transition into another state.

Since $\theta^{*} \{ A(m_{1} )\}$, $\theta^{*} \{ A(m_{2} )\}$, $\theta^{*} \{ A(.)\}$, $\theta^{*} \{ A(m_{t} )\}$, $\theta^{*} \{ A(..)\}$, and $\theta^{*} \{ A(m_{T} )\}$ are all exhaustive steady-state probabilities corresponding to states $A(m_{1} )$, $A(m_{2} )$, $A(.)$, $A(m_{t} )$, $A(..)$, and $A(m_{T} )$, respectively, the following equation can also be used:

14 $$ \theta^{*} \{ A(m_{1} )\} + \theta^{*} \{ A(m_{2} )\} + \theta^{*} \{ A(.)\} + \theta^{*} \{ A(m_{t} )\} + \theta^{*} \{ A(..)\} + \theta^{*} \{ A(m_{T} )\} = 1 $$

Equation (14) ensures that all exhaustive steady-state probabilities are captured.

If *c{A(m*_*1*_*)}*, *c{A(m*_*2*_*)}*, *c{A(.)}*,..*C{A(m*_*t*_*)*, *C{A(..)}..C{A(m*_*T*_*)* represent the average costs of treatment corresponding to states *A(m*_*1*_*)*, *A(m*_*2*_*)*, *A(.)*,..*A(m*_*t*_*)*, *A(..)*, *A(m*_*T*_*)*, respectively, then the deterministic long-term average cost of treatment per patient can be ascertained as per Eq. (15):

15 $$ \begin{aligned} E^{\det } (c) & = [c\{ A(m_{1} )\} \times \theta_{{}}^{*} \{ A(m_{1} )\} + c\{ A(m_{2} )\} \times \pi_{{}}^{*} \{ A(m_{2} )\} + c\{ A(.)\} \times \theta_{{}}^{*} \{ A(.)\} \\ & \quad { + }c\{ A(.)\} \times \theta_{{}}^{*} \{ A(.)\} + c\{ A(m_{t} )\} \times \theta_{{}}^{*} \{ A(m_{t} )\} + c\{ A(..)\} \times \theta_{{}}^{*} \{ A(..)\} \\ & \quad { + }c\{ A(m_{T} )\} \times \theta_{{}}^{*} \{ A(m_{T} )\} ] \\ \end{aligned} $$

If *D(1)*, *D(2)*, *D(.)*, *D(k)*, *D(..)*, and *D(K)* are the various treatment decisions, then these treatment decisions could be mapped with individual states of the medical condition in binary terms, as shown in Table 3(c). It is to be noted that any element of this matrix has binary properties such that *D(k,m*_*t*_*)* ϵ (0,1). The rationale for this is that depending on the actual state of a medical condition, different treatment decisions can be taken depending on the patient. For instance, for a “minor” cardiovascular condition, a relatively healthy patient’s treatment might be accompanied by non-surgical and lifestyle change-oriented approaches. On the other side, for a “minor” cardiovascular condition for a patient with comorbidities, a moderate surgical procedure with some medicinal interventions might be more appropriate. However, the flip side to a deterministic policy of a treatment decision is that the medical service provider would not have any leeway to consider different treatment decisions corresponding to different states of a medical condition in a commensurate manner. Therefore, a probability distribution should be employed to map a particular treatment decision with a certain medical condition state.

##### Randomized policy

A randomized policy matrix with the mapping of states of a medical condition and decision is shown in Table 3(d). For a given medical condition state *A(m*_*T*_*)* and treatment decision *D(k)*, let *y(k*, *m*_*t*_*)* be a steady-state unconditional probability, which can be interpreted as the following:

16 $$ y(k,m_{t} ) = p\{ state \, = \, A(m_{t} ) \, and \, decision \, = \, k\} $$

The abovementioned steady-state unconditional probability holds the form of a joint probability containing both the state of a medical condition and the medical decision corresponding to that state.

Following the theory of conditional probability, each *y(k*, *m*_*t*_*)* is closely related to *D{k*, *m*_*t*_*)* such that the following mathematical expression formulated in Eq. (17) would be satisfied:

17 $$ y(k,m_{t} ) = \theta^{ * } \{ A(m_{t} )\} \times D(k,m_{t} )\} $$

18 $$ {\text{such}}\,{\text{that}}\quad \theta^{ * } \{ A(m_{t} )\} = \sum\limits_{k = 1}^{K} {y(k,m_{t} )} $$

19 $$ {\text{and}}\,{\text{so}}\,{\text{that}}\quad D(k,m_{t} ) = \frac{{y(k,m_{t} )}}{{\sum\nolimits_{k = 1}^{K} {y(k,m_{t} )} }} $$

Equations (18) and (19) capture the constraints related to steady-state probabilities.

There exist three sets of constraints for $y(k,m_{t} )$.

20 $$ {\text{(a}})\quad \theta^{*} \{ A(m_{1} )\} + \theta^{*} \{ A(m_{2} )\} + \theta^{*} \{ A(.)\} + \theta^{*} \{ A(m_{t} )\} + \theta^{*} \{ A(..)\} + \theta^{*} \{ A(m_{T} )\} = 1 $$

21 $$ {\text{so}}\,{\text{that}}\quad \sum\limits_{{m_{1} }}^{{m_{T} }} {\sum\limits_{k = 1}^{K} {y(k,m_{t} } } ) = 1 $$

Equations (20) and (21) ensure that the sum of all exhaustive steady-state probabilities and steady-state unconditional probabilities is equal to 1.

(b) From the results on steady-state probabilities, the following can be formulated:

22 $$ \theta (s) = \sum\limits_{{m_{1} }}^{{m_{T} }} {\theta^{*} } \{ A(m_{t} )\} \times p_{k} \{ A(m_{1} ) \to A(m_{t} )\} $$

23 $$ \begin{aligned} & \sum\limits_{k = 1}^{K} {y(s,m_{t} } ) = \sum\limits_{{m_{1} }}^{{m_{T} }} {\sum\limits_{k = 1}^{K} {y(k,m_{t} } } ) \times p_{k} \{ A(m_{1} ) \to A(m_{t} )\} \, \\ & \quad for\quad s = \{ A(m_{1} ),A(m_{2} )...A(m_{t} )...A(m_{T} )\} \\ \end{aligned} $$

24 $$ ({\text{c}})\quad 0 \le y(k,m_{t} ) \le 1,\,\,for\,\,k = \, 1, \, 2, \, 3, \ldots .K\,\,\,and\,\,m_{1} , \, m_{2} , \, \ldots m_{t} \ldots .m_{T} $$

Equation (24) ensures that the steady-state unconditional probability has a bound of 0 and 1.

Hence, the long-term expected cost $E^{Rand} (c)$ per patient can be given by the following expression:

25 $$ E^{Rand} (c) = \sum\limits_{{m_{1} }}^{{m_{T} }} {\sum\limits_{k = 1}^{K} {\theta^{*} } } \{ A(m_{t} )\} \times c\{ A(m_{t} )\} \times D(k,m_{t} ) = \sum\limits_{{m_{1} }}^{{m_{T} }} {\sum\limits_{k = 1}^{K} {c\{ A(m_{t} )\} \times y(k,m_{t} } } ) $$

Equation (25) expresses the expected total cost under the randomized policy.

Hence, the linear programming model chooses *y(k,m*_*t*_*)* so as to

26 $$ Minimize\left\{ {\sum\limits_{{m_{1} }}^{{m_{T} }} {\sum\limits_{k = 1}^{K} {c\{ A(m_{t} )\} \times y(k,m_{t} )} } } \right\} $$

Equation (26) signifies the objective function considering the costs at each state and the corresponding probabilistic basic variable.

This is subject to the constraint

27 $$ (a)\quad \sum\limits_{{m^{1} }}^{{m_{T} }} {\sum\limits_{k = 1}^{K} {y(k,m_{t} } } ) = 1 $$

Equation (27) captures the fact that the sum of all exhaustive steady-state unconditional probabilities is equal to 1.

28 $$ (b)\quad \sum\limits_{k = 1}^{K} {y(k,m_{t} } ) - \sum\limits_{{m_{1} }}^{{m_{T} }} {\sum\limits_{k = 1}^{K} {y(k,m_{t} } } ).p_{k} \{ A(m_{1} ) \to A(m_{t} )\} = 0{\text{, for }}m_{1}^{{}} ,m_{2} ,...m_{t} ,...m_{T} $$

Equation (28) establishes the interrelation between steady-state unconditional probabilities and mean transition probabilities.

29 $$ ({\text{c}})\quad 0 \le y(k,m_{t} ) \le 1,{\text{ for m}}_{1} ,m_{2} ...m_{t} ...m_{T} ;k = 1,2,3....K $$

Equation (29) ensures that all unsteady-state unconditional probabilities are bounded between 0 and 1.

**Fig. 3:**
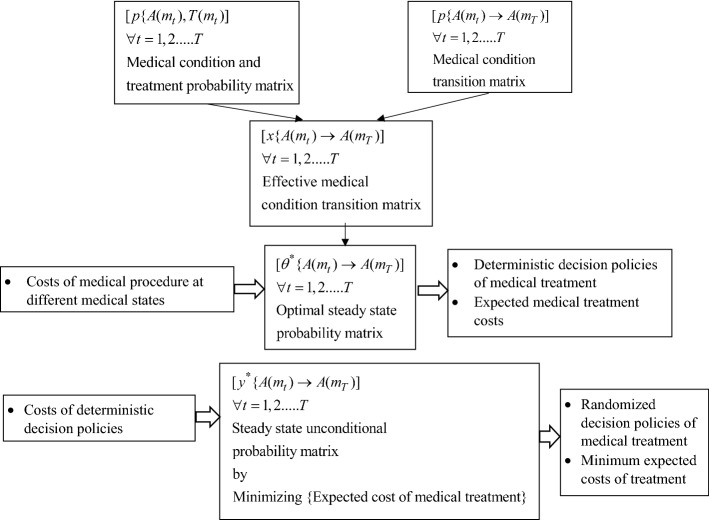
Schema of the modeling stage

The model represented by mathematical Eqs. (26)–(29) represents the linear programming (LP) model containing *(M*_*T*_ + *2)* functional constraints and *K(M*_*T*_ + *1)* decision variables. Because the above LP model can be solved using commercial solvers, once the $y(k,m_{t} )$ values are obtained, each $D(k,m_{t} )$ can be ascertained. Figure 3 depicts the step-by-step schema of the modeling phase. An important advantage of the developed model based on the randomized policy is that though a simplistic optimal medical policy can be found under the deterministic policy based on the simplex method as the optimization algorithm, the randomized policy aids in improving the optimal setting in that costs can also be mitigated considering the mapping of respective decisions concerning pertinent medical condition states. Further, the randomized policy also plays a useful role in that by converting integer variables {$D(k,m_{t} )$ s} to continuous variables {$y(k,m_{t}$)s}, we ensure that linear models and accompanying optimization algorithms can work. This is especially important given that the analogy in integer programming is to use the LP relaxation so that the simplex method can be applied, and then the integer property solutions can hold so that the optimal LP relaxation solution turns out to be integer anyway. A Markovian decision process (MDP) and other formulations and accompanying methods such as dynamic programming and reinforcement learning also focus on transitions from one state to another. However, our prescriptive models have several advantages over other approximation-based techniques such as approximate dynamic programming. First, as opposed to approximate dynamic programming (wherein no standard mathematical formulation can be obtained in that each mathematical formulation has its own characteristics depending on the problem structure), our evolved formulation under both the deterministic and randomized policy is anchored to the linear model. One significant advantage of this is that we can achieve the exact optimal solution(s) instead of the near-optimal solution(s) in the case of approximation-based techniques. Second, as opposed to approximate dynamic programming (which makes a series of interrelated decisions), in our formulations, we are able to represent the problem in terms of linear models such that aggregate level optimal solution(s) can be obtained, leading to the merits of both parametrization and scalability. Third, as opposed to approximate dynamic programming, which relies on exhaustive computational enumeration, a technique that often becomes problematic when the number of states increases, our model does not suffer from this in that, irrespective of the increase in the number of states, the underlying solution algorithm would still comprise linear model-based algorithms. Thus, due to its high degree of parametrization, scalability, and anchoring in linear modeling, the proposed models ensure optimal treatment policies. The corresponding costs can be converged on time (instead of non-polynomial times for many non-linear and approximation-based models).

## Stepwise solution methodology with an illustrative example

In order to illustrate our methodology, we consider the data obtained from 115 clinicians (experts) who had been affiliated with a multi-specialty hospital chain in Eastern India as cardiovascular specialists and are now retired. These hospital chains are part of a large private multi-specialty hospital chain headquartered in the capital city of an Eastern Indian state. Being a reputable hospital in Eastern India, patients from both the native state and other states often flock to this hospital due to its affordable health care costs. To preserve anonymity, we do not explicitly mention the names of the hospital chain and 115 clinicians. The pertinent data related to the study and experts’ profiles are listed in Table 9 in the “Appendix” section. Referring to Table 9, these experts were either DMs or MDs (doctors of medicine) and had been associated with the said hospital chain work-wise for a minimum of 22 years and a maximum of 34 years. These clinicians possessed the expertise to diagnose, treat, and prevent cardiovascular diseases, with experience in both non-invasive and surgical procedures.

### Qualitative stage

#### Information gathering and questionnaire phase

A belief about certainty (or uncertainty) by an expert about some state of a medical condition is often grounded in data or experience gained during professional practice, or both (Constantinou et al., 2016). While expert judgment can be useful when information is often incomplete, experts can still make some mistakes (Hemming et al., 2018). Therefore, to mitigate the effects of such mistakes, we employed the IDEA protocol of expert elicitation in particular (a form of the modified Delphi method), which comprised four sequential steps (i.e., “Investigate”, “Discuss”, “Estimate”, and “Aggregate;” Hemming et al., 2018). During the “Investigate” step, all experts individually answered questions and provided reasons for their respective judgments. Following this, during the “Discuss” step, experts were shown anonymized responses from other experts and a visual summary of responses. In the “Estimate” step, all experts made a final and private estimate. In the final “Aggregate” step, the means of experts’ second-round responses were determined. Experts were permitted to review these calculations, add commentary and correct residual misunderstandings.

The 115 experts (clinicians) opined that they had typically observed four major states of cardiovascular conditions, which often form the basis for the corresponding level of medical diagnosis and treatment. These levels also form the basis for corresponding administrative procedures, including those of the hospital and insurance companies (in cases wherein the patients were insured). Table 4 denotes this classification of the four states of cardiovascular conditions.

**Table 4:** Thematic output related to states of cardiovascular conditions along with related attributes

States of the medical condition	Surgical intervention(s)	Medicinal intervention(s)	Other considerations
Minor	No surgery is often needed	Some medicines may be prescribed	Emphasis on physical exercise for recovery
Moderate	Minor surgeries are often needed	Significant medicinal usage	Recovery time may take a few weeks
Major	Major surgical procedures needed	Heavy medicinal usage	Recovery time may take a few months
Severe	Major surgical procedures with significant risks (many times after heart attack)	Heavy medical usage	Recovery times may take more than 6 months and, in certain cases, even a year

Referring to Table 4, thematically, there are four major cardiovascular states, viz. “Minor”, “Moderate”, “Major”, and “Severe.”. However, it is important to note that though we would consider these major states as different discrete levels corresponding to the medical conditions, from the perspective of a medical practitioner, these different states essentially signify an increasing level of severity in the medical condition’s continuum. The semi-structured interviews identified another set of important themes: whether these four corresponding states typically accompany any surgical or medicinal interventions. Finally, some nuances related to recovery were also captured based on the semi-structured interviews.

Thereafter, the clinicians were asked to fill out a Google form-based survey created to capture the inputs related to cardiovascular conditions during the questionnaire phase. The survey essentially captured three broad dimensions. First, what were the typical frequencies of reported diagnosis of the four states of the medical condition? Second, what were the frequencies of the accuracy of medical treatment corresponding to each of the four states of the diagnosed condition? Third, what were and to what extent did the transitions from a particular state of medical condition impact itself and subsequent degenerative states? Table 10 in the “Appendix” provides an excerpt of the questionnaire developed. We specifically emphasized data from the last three years since the hospital chain had ensured the linking of individual cases with patients' personalized Aadhaar numbers (Unique Identification Authority of India). Further, it is important to note that all clinicians did not want to report the actual numbers; rather, they were comfortable reporting the percentages. Therefore, we relied on frequencies.

#### Statistical testing and validation

**Fig. 4:**
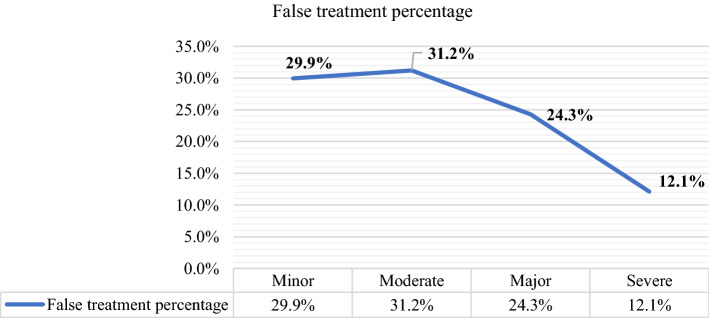
Frequencies and false treatment for the four states of the cardiovascular condition

When we observed the reported frequencies of the four states of a diagnosed cardiovascular condition, we ascertained that out of 115, 13 clinicians reported that frequencies were outliers. In the case of the remaining 102 clinicians’ data, there was a clear pattern in the reported frequencies. This pattern manifested clearly in that frequency of diagnosis of the “minor” state was greater than that of the “moderate” state, which in turn was greater than that of the “major” state. Finally, the “severe” state had the lowest reported frequencies. Therefore, we only considered the inputs of 102 clinicians. In particular, one of the primary variables of interest was the mean probability of a diagnosed state (based on the mean frequency of 102 considered clinicians). Figure 4 reports the mean frequencies of false treatment corresponding to each state of the medical condition.

However, before assuming that we could obtain and use these mean values for the subsequent modeling stage, it was essential to ascertain whether the mean frequency (probability) of a particular state of diagnosis was statistically the same across the 102 clinicians considered (within a cutoff difference of 2%, as advised by the few senior clinicians in the study). Before performing this, we examined the normality of sample data corresponding to each state of diagnosis, and we tested for normality of the sample at a 95-percent confidence interval. We first laid out the data graphically and looked at the histogram and Q-Q plots, which indicated a normal distribution. Further, we also performed the Shapiro–Wilk test for the reported probabilities. In this test, the *p-*value was greater than 0.05, indicating that the probabilities corresponding to each state of diagnosis were approximately normally distributed. Thereafter, we carried out an analysis of variance (ANOVA) one-factor test at *p* < 0.05 for the data from the 102 clinicians (leaving aside the outlier data from 13 clinicians), where the following hypothesis represented the null hypothesis:

H(0): Reported frequencies of diagnosed states remain statistically the same for the twelve clinicians within a cutoff percentage of 2.

Based on the *p-value* and *t* statistic, we failed to reject the null hypothesis of frequencies at a 95-percent confidence level.

In order to test the order of the mean probabilities of the reported states (such that the mean probability of diagnosis at a minor state was greater than that at a moderate state and so forth), the following null hypothesis was postulated.

H(0): The reported frequencies of the diagnosed states are such that order remains: frequency of “Minor” state > frequency of “Moderate” state > frequency of “Major” state > frequency of “Severe” state for all 102 clinicians.

In order to test the aforementioned hypothesis, a total of six pair-wise two-tailed *t*-tests were carried out at a 95-percent confidence level (i.e., *p* < 0.05). For the sake of brevity, we report two such comparisons, which are provided in Table 5: one related to “Minor” versus “Moderate”, while the other pertains to “Minor” versus “Major.”

**Table 5:** Snapshot of pairwise two-tailed *t*-test results

	Minor	Moderate
*(a) Comparison of frequency of the states “Minor” and “Moderate”*		
Mean	0.54366667	0.31075
Observations	102	102
Pearson Correlation	− 0.21411211	
t Stat	− 0.04093975	
P(T ≤ t) one-tailed	0.484038782	
t Critical one-tailed	1.795884819	
P(T ≤ t) two-tailed	0.968077565	
t Critical two-tailed	2.20098516	

	Minor	Major
*(b) Comparison of frequency of the states “Minor” and “Major”*		
Mean	0.54366667	0.117333
Observations	102	102
Pearson Correlation	− 0.1847954	
t Stat	0.1405299	
P(T ≤ t) one-tailed	0.44539083	
t Critical one-tailed	1.79588482	
P(T ≤ t) two-tailed	0.89078165	
t Critical two-tailed	2.20098516	

Based on the *p-*value and *t* statistic, it was verified that the mean probability of diagnosis at the minor stage > the mean probability of diagnosis at the moderate state > the mean probability of diagnosis at the major state > the mean probability of diagnosis at the severe state. We also concluded that the frequencies (probabilities) of finding a particular state of the cardiovascular condition remained statistically the same for the 102 clinicians. Therefore, we used the mean medical condition probabilities *p{A(m*_*t*_*)*, *T(m*_*t*_*)}s* and mean transition probabilities $p\{ A(.) \to A(m_{t} )\}$ s for the subsequent modeling stage.

Notably, all statistical tests performed to validate the postulated hypotheses were parametric in nature. There were inherent assumptions about the population parameters from which the 102 relevant (and workable) samples were drawn. Since we were also interested in the important nuances related to the sample data (such as the order of reported frequencies at each state), we naturally preferred parametric tests over non-parametric tests, even though parametric tests often require normality tests and non-parametric tests do not necessarily require such tests on the sample.

### Modeling stage

#### Populate the medical condition and transition matrices-$[p\{ A(m_{t} ),T(m_{t} )]$ and $[p\{ A(m_{t} ) \to A(m_{T} )]$

Table 6(a) lists the mean values of the $[p\{ A(m_{t} ),T(m_{t} )]$ matrix.

**Table 6:** Pertinent data related to the case example

State of medical condition	Treated as			
	Minor	Moderate	Major	Severe
*(a) Mean medical condition and treatment probability matrix as determined based on expert inputs* $[p\{ A(m_{t} ),T(m_{t} )]$				
Minor	0.701	0.160	0.097	0.042
Moderate	0.071	0.688	0.127	0.114
Major	0.100	0.112	0.757	0.031
Severe	0.000	0.020	0.101	0.879

State	Minor	Moderate	Major	Severe
*(b) Mean medical condition transition values based on expert inputs* $[p\{ A(m_{t} ) \to A(m_{T} )]$				
Minor	0.6	0.25	0.1	0.05
Moderate	0	0.58	0.227	0.15
Major	0	0	0.65	0.35
Severe	1	0	0	0
*(c) Effective medical condition transition matrix* $[x\{ A(m_{t} ) \to A(m_{T} )]$				
Minor	0.890	0.085	0.021	0.004
Moderate	0.000	0.897	0.065	0.038
Major	0.000	0.000	0.978	0.022
Severe	1.000	0.000	0.000	0.000

Referring to Table 6(a), the highest percentages of cases pertained to the “minor” cardiovascular condition, while the lowest percentage pertained to the “severe” condition. Further, in the case of an actual medical condition being “severe”, the percentage of false treatment was the lowest.

Table 6(b) lists the mean transition probabilities associated with the four states. It is also worth mentioning that these probabilities were the best case judgments from the 102 clinicians as per their professional understanding and experience.

#### Determine effective medical condition transition matrix- $[x\{ A(m_{t} ) \to A(m_{T} )]$

Using the values given in Table 6(a) and (b), the elements of the medical condition transition matrix were determined using Eqs. (7) and (8).

For instance, *p (minor)* was determined as follows (transition to own state):

30 $$ {\text{p}} (minor) = \frac{0.701 \times 0.6}{{(0.701 \times 0.6 + 0.160 \times 0.25 + 0.097 \times 0.1 + 0.042 \times 0.05)}} $$

Another instance of transition to a different state—that is, ${\text{p(minor}} \to {\text{moderate)}}$—is illustrated below.

31 $$ {\text{p(minor}} \to {\text{moderate) = }}\frac{0.160 \times 0.25}{{(0.701 \times 0.6 + 0.160 \times 0.25 + 0.097 \times 0.1 + .042 \times 0.05)}} $$

Along similar lines, the remaining elements of the matrix $[x\{ A(m_{t} ) \to A(m_{T} )]$ were determined and are listed in Table 6(c).

#### Ascertain optimal state probability matrix along with the deterministic decision policies

If $\theta {\text{(minor)}}$, $\theta {\text{(moderate)}}$, $\theta {\text{(major)}}$, and $\theta {\text{(severe)}}$ are the long-term steady-state probabilities corresponding to the states “minor”, “moderate”, “major”, and “severe,” respectively, then the following set of simultaneous equations can be written:

32 $$ \begin{aligned} & \theta^{*} ({\text{minor}}) = 0.890 \times \, \theta^{*} ({\text{minor}}) + \theta \times \theta^{*} ({\text{minor}}) \\ & \theta^{*} ({\text{moderate}}) = 0.085 \times \, \theta^{*} ({\text{minor}}) + \, 0.897 \times \, \theta^{*} ({\text{moderate}}) \\ & \theta^{*} ({\text{major}}) = 0.021 \times \, \theta^{*} ({\text{minor}}) + 0.065 \times \, \theta^{*} ({\text{moderate}}) + \, 0.978 \times \, \theta^{*} ({\text{major}}) \\ & \theta^{*} ({\text{severe}}) = 0.004 \times \, \theta^{*} ({\text{minor}}) + 0.038 \times \, \theta^{*} ({\text{moderate}}) + \, 0.22 \times \, \theta^{*} ({\text{major}}) \\ & \theta^{*} ({\text{minor}}) + \, \theta^{*} ({\text{moderate}}) + \, \theta^{*} ({\text{major}}) + \, \theta^{*} ({\text{severe}}) = 1 \\ \end{aligned} $$

Solving the set of equations given in Eq. (32) on ILOG CPLEX Optimization Studio yielded the following steady-state values:

$$ \theta^{*} {\text{(minor) = 0}}{.171; }\theta^{*} {\text{(moderate)}} = { 0}{\text{.158; }}\theta^{*} {\text{(major) = }}{.650; }\theta^{*} {\text{(severe) = 0}}{.021} $$

The treatment costs corresponding to each of the four medical states (i.e., “minor”, “moderate”, “major” and “severe”) were $570, $1590, $6500, and $13,500, respectively, as determined by the average costs of the previous three years. Considering these costs and the optimal steady-state probabilities, and using Eq. (15), *E*^*Det*^*(c)* was determined to be $4859.84.

Based on the clinicians’ inputs and their professional experience, Table 7(a) denotes the major decisions that pertained to each of the four states.

**Table 7:** Decision description and mapping for randomized policy

Notation of decision	Action	Relevant states
*(a) Decision, corresponding action, and relevant states*		
D1	Patients are advised for regimen and lifestyle changes with minimal medicinal interventions	Minor, Moderate
D2	Patients are put on a medical regimen with major lifestyle changes under a specialist’s supervision and follow-ups	Minor, Moderate, Major
D3	Patients typically undergo a minor surgical procedure with few post-surgery precautions	Moderate, Major, Major
D4	Patients typically undergo a major surgical procedure with major post-surgery precautions and a major medicinal regimen	Major, Severe
D5	Patents typically undergo a series of major surgical procedures with major post-surgery precautions and a medicinal regimen that goes for months	Severe

State	*D1*	*D2*	*D3*	*D4*	*D5*
*(b) Decision mapping*					
Minor	*y(1,1)*	–	–	–	–
Moderate	*y(1,2)*	*y(2,1)*	*y(3,2)*		
Major	–	*y(2,2)*	*y(3,3)*	*y(4,3)*	
Severe	–	*y(2,3)*	*y(3,4)*	*y(4,4)*	*y(5,4)*

It is worth mentioning that these decisions had historically been associated with the corresponding states of cardiovascular conditions. When we inquired why a particular decision seemed to map with a couple of states (e.g., D1 was associated with both “minor” and “moderate” states), the clinicians revealed that decisions about a specific medical condition also have certain subjectivities and, therefore, there cannot always be clear one-to-one mapping. The experts reasoned that other dimensions related to patients, such as comorbidities (glucose level, hypertension), the extent of physical fitness, and a history of specific medical conditions, also play a major role in warranting one decision over another.

#### Ascertain randomized policies

Using the information provided in Table 7(a) and the convention as formalized in Table 3(d), the steady-state unconditional probabilities, *y(k*,* m*_*t*_*)*s, were determined. The mapping of these variables with the state of medical conditions and corresponding decisions is illustrated in Table 7(b). Using Eq. (26), therefore, the objective function would be the minimization of expected costs under a randomized policy such that *E*^*Rand*^*(c)* can be expressed as follows:

33 $$ \begin{aligned} E^{Rand} (c) & = Minimize\{ 50y(1,1) + { 570}y(1,2) + \, 570y(2,1) + { 570}y(2,2) + { 1590}y(2,3) \\ &\quad + 1590y(3,2) + { 1590}y(3,4) + \, 6500y(4,3) + { 13,500}y(4,4){ + 13,500}y(5,4){\text{\} }} \\ \end{aligned} $$

It is worth mentioning that, in the above equation, though decision D1 corresponding to the “minor” state does not have a clear treatment/medical cost, as per the description of this decision in Table 7(a), nonetheless certain administrative costs in the order of $50 are typically incurred.

Following this convention, as formalized by Eq. (27), the following can be expressed:

34 $$ y(1,1) + \, y(1,2) + \, y(2,1) + y(2,2) + y(2,3) + y(3,2) + y(3,4) + y(4,3) + y(4,4) + y(5,4) = 1 $$

Corresponding to Eqs. (28) and (29), there would be 4 (hard constraints) and 22 (soft constraints). Among the 22 soft constraints, 11 were greater than equal to 0, and the remaining 11 were less than equal to 1.

The developed mathematical model belongs to linear programming and was solved using ILOG CPLEX Optimization Studio. The following results were obtained:

$$ \begin{aligned} & \left\{ {{\text{y}}\left( {{1},{1}} \right),{\text{ y}}\left( {{1},{2}} \right)} \right\} \, = \, \left\{ {0.{136}, \, 0} \right\}; \, \left\{ {{\text{y}}\left( {{2},{1}} \right),{\text{ y}}\left( {{2},{2}} \right),{\text{ y}}\left( {{2},{3}} \right)} \right\} \, = \, \left\{ {0, \, 0.{215}, \, 0} \right\}; \\ & \left\{ {{\text{y}}\left( {{3},{2}} \right),{\text{ y}}\left( {{3},{3}} \right),{\text{ y}}({3},{4}} \right\} \, = \, \left\{ {0, \, 0.{435}, \, 0} \right\}; \, \left\{ {{\text{y}}\left( {{4},{3}} \right),{\text{ y}}\left( {{4},{4}} \right)} \right\} \, = \, \left\{ {0.0{97}, \, 0} \right\}; \, \left\{ {{\text{y}}\left( {{5},{4}} \right)} \right\} = \left\{ {0.{117}} \right\} \\ \end{aligned} $$

Using equation number 19, the following *D(k,m*_*t*_*)*s were determined:

$$ \begin{aligned} & \left\{ {{\text{D}}\left( {{1},{1}} \right),{\text{ D}}\left( {{1},{2}} \right)} \right\} \, = \, \left\{ {{1}, \, 0} \right\}; \, \left\{ {{\text{D}}\left( {{2},{1}} \right),{\text{ D}}\left( {{2},{2}} \right),{\text{ D}}\left( {{2},{3}} \right)} \right\} \, = \, \left\{ {0,{ 1}, \, 0} \right\}; \\ & \left\{ {{\text{D}}\left( {{3},{2}} \right),{\text{ y}}\left( {{3},{3}} \right),{\text{ y}}({3},{4}} \right\} \, = \, \left\{ {0,{ 1}, \, 0} \right\}; \, \left\{ {{\text{D}}\left( {{4},{3}} \right),{\text{ D}}\left( {{4},{4}} \right)} \right\} \, = \, \left\{ {{1}, \, 0} \right\}; \, \left\{ {{\text{D}}\left( {{5},{4}} \right)} \right\} = \left\{ {1} \right\} \\ \end{aligned} $$

*E*^*Rand*^*(c)* was determined to be $3031.42.

Comparing the results from both the deterministic and randomized policy produced a couple of important points. First, as opposed to any lack of mapping of the pertinent decision policy for the various states of the medical condition, the randomized policy resulted in the steady-state probabilities and the mapping of the pertinent decision policy with respect to the various states of the medical condition. Second, the expected cost of treatment under the randomized policy was superior to that of the deterministic policy, indicating there might be a preference for the randomized medical treatment policy over a long horizon from an economic standpoint. Finally, the randomized policy was slightly more predisposed than the deterministic policy toward dealing with severe states of the medical condition, considering that the steady-state probability in the case of the randomized policy was higher than that of the deterministic policy.

## Key findings and analysis

In this section, we thematically discuss the important findings and accompanying nuances related to both stage 1 and stage 2 of the study.

### Findings and analyses related to the qualitative stage (stage 1)

#### Homogeneity of medical condition probabilities

The data reported by the remaining 102 clinicians were fairly homogenous in that the probability of the cardiovascular condition remaining in any particular state, as reported by 102 of the 115 clinicians, lay within the 2% range. An important reason for this relative homogeneity is that most of these clinicians belonged to the same facility of the hospital chain, while several others belonged to a different facility (in a different city) within the state. Post-study follow-up discussions with clinicians showed that the vast majority (more than 85%) of the patients belonged to the same geographic regions (i.e., same state) and were predominantly aged 55 or over. Further, a fairly low and uniform population sample size also supports the relative homogeneity of the probabilities of different states remaining in a narrow range.

#### False treatment probabilities

Further, referring to Fig. 4, it can be said that perhaps the minimal error in judgment on the part of the healthcare service provider is related to the diagnosis and treatment of a “severe” state. This finding is fairly intuitive in that patients’ symptoms, and conditions related to more severe states of a cardiovascular condition are much easier to diagnose as opposed to those of less severe states. The two senior-most clinicians also validated this. However, this is because medical practitioners do not look at states of such conditions in terms of different discrete levels; instead, they consider different states as a part of the severity continuum of the condition. When the two senior-most clinicians were probed with respect to the broad reasons for almost 30% of false treatments in both “minor” and “moderate” states, an important systemic reason was given by the two clinicians. The reason pertained to gaps in the case history (even though pertinent case history was being captured through the unique identification of the individual patients) in the majority of the patient cases. The barriers of the non-availability of previous diagnosis and treatment, a lack of interoperability, and lack of integration with the data of those patients who had prior treatment at government facilities had a negative effect, as a fairly significant number of patients received inaccurate medical treatment at each of the states of the medical condition based on the professional feedback from the clinicians. These systemic issues playing a role in jeopardizing the diagnosis and treatment efforts in terms of escalated costs and poor health outcomes have been adequately captured by Dhagarra et al. (2019).

#### Transition probabilities

Referring to Table 6(c), it is fairly intuitive that the highest probability value corresponds to the “minor” state remaining in this state itself, with the least probability value corresponding to a transition from “minor” to “severe.” The probabilistic values of the transitions from “minor” to “moderate” and from “minor” to “major” remained in an intermediate range. However, a counter-intuitive finding here pertains to the fact that the probabilistic value of the transition from “moderate” to “severe” was higher compared with the transition from “major” to “severe.” For this observation, we again probed the two senior-most clinicians. It was revealed that a large part of this could be attributed to the fact that, in many instances, it was found that patients who did not have any surgery and were associated mostly with a “moderate” state did not often adhere to the post-treatment protocols and lifestyle changes that were recommended.

### Findings and analyses related to modeling stage (stage 2)

#### Comparison of deterministic and randomized policy

A comparison of the randomized policy and deterministic policy of medical treatment revealed that the expected long-term cost of treatment in the case of the randomized policy was significantly lower than that of the deterministic policy. An important reason for this difference is that in the case of the randomized policy, the optimal steady-state unconditional probability is less skewed toward the “severe” state than a deterministic policy, which is more skewed toward the “severe” state. The cost of diagnosis, treatment, surgeries, and post-treatment in a “severe” state was found to be significantly higher in the case of a “severe” state as opposed to the other states. Because the treatment decision in the deterministic policy revolves around the healthcare service provider prescribing an appropriate level of treatment depending on the history of the case, a certain expected cost of the medical treatment would result. On the other hand, the randomized policy revolves around an attempt by the service provider in such a manner that certain acceptable treatment decisions can be mapped to individual states of a medical condition such that the expected treatment costs can be minimized. The adoption of randomized medical treatment policies can be of particular value to large developing countries such as India, which are often characterized by limits on governmental spending, inadequate healthcare infrastructure (relative to developed countries), and variable quality of healthcare (Kong et al., 2018). However, this is not to suggest that a randomized policy that optimizes the long-term treatment costs is universally superior to a deterministic policy and should always be adopted. Of particular emphasis would be how the treatment outcomes of the two policies contrast with each other in that if the instances of false treatment in the case of the randomized policy are not too different compared with the deterministic policy, the randomized policy can be an acceptable lever for keeping the treatment costs lower.

#### Sensitivity analysis

To understand the impact of the changes in the steady-state probabilities on the total expected treatment cost spread over time, we conducted a sensitivity analysis for the deterministic policy. In particular, we varied the steady-state probability corresponding to the states “minor”, “moderate”, “major”, and “severe” one at a time within ± 10%. Table 8 represents these settings. Further, the sensitivity analysis was performed under higher- and lower-cost conditions. Referring to Table 8(a) and considering, for example, when *θ*(minor) is varied from 90 to 100% of its optimal value, the higher side of the expected cost would result when *θ*(moderate) and *θ*(major) remains constant, and *θ*(severe) varies based on variation in *θ*(minor; due to the magnitude of associated cost coefficients corresponding to the probabilities of an individual state). When *θ*(minor) is varied from 101 to 110% of its optimal value, a higher side of the expected cost will result when *θ*(major) and *θ*(severe) remain constant, and π(moderate) varies based on variation in *θ*(minor). When *θ* (minor) is varied from 90 to 100% of its optimal value, the lower side of the expected cost will result when *θ*(major) and *θ*(severe) remain constant, and θ(moderate) varies based on variation in *θ*(minor) (due to the associated cost coefficients corresponding to the probabilities of the individual state). When *θ*(minor) is varied from 101 to 110% of its optimal value, the lower side of the expected cost will result when *θ*(major) and *θ*(major) remain constant, and *θ*(severe) varies based on variation in π(minor). Table 8 lists all possible instances of variations under both higher and lower-cost conditions and considers the range of variation, i.e., from 90 to 110%.

**Table 8:** Conditions for the sensitivity analysis

Range of variation	Variable subject to variation (A)	Variables remaining constant (B)		Variable varying due to variation in A
*(a) Under higher-cost conditions*				
(90% to 100%) variation in variable of interest	*θ*(minor)	*θ*(moderate)	*θ*(major)	*θ*(severe)
	*θ*(moderate)	*θ*(minor)	*θ*(major)	*θ*(severe)
	*θ*(major)	*θ*(minor)	*θ*(moderate)	*θ*(severe)
	*θ*(severe)	*θ*(minor)	*θ*(moderate)	*θ*(major)
(101% to 110%) variation in variable of interest	*θ*(minor)	*θ*(major)	*θ*(severe)	*θ*(moderate)
	*θ*(moderate)	*θ*(major)	*θ*(severe)	*θ*(minor)
	*θ*(major)	*θ*(moderate)	*θ*(severe)	*θ*(minor)
	*θ*(severe)	*θ*(moderate)	*θ*(major)	*θ*(minor)
*(b) Under lower-cost conditions*				
(90% to 100%) variation in variable of interest	*θ*(minor)	*θ*(major)	*θ*(severe)	*θ*(moderate)
	*θ*(moderate)	*θ*(major)	*θ*(severe)	*θ*(minor)
	*θ*(major)	*θ*(moderate)	*θ*(severe)	*θ*(major)
	*θ*(severe)	*θ*(moderate)	*θ*(major)	*θ*(minor)
(101% to 110%) variation in variable of interest	*θ*(minor)	*θ*(moderate)	*θ*(major)	*θ*(severe)
	*θ*(moderate)	*θ*(major)	*θ*(minor)	*θ*(severe)
	*θ*(major)	*θ*(minor)	*θ*(moderate)	*θ*(severe)
	*θ*(severe)	*θ*(moderate)	*θ*(minor)	*θ*(major)

**Fig. 5:**
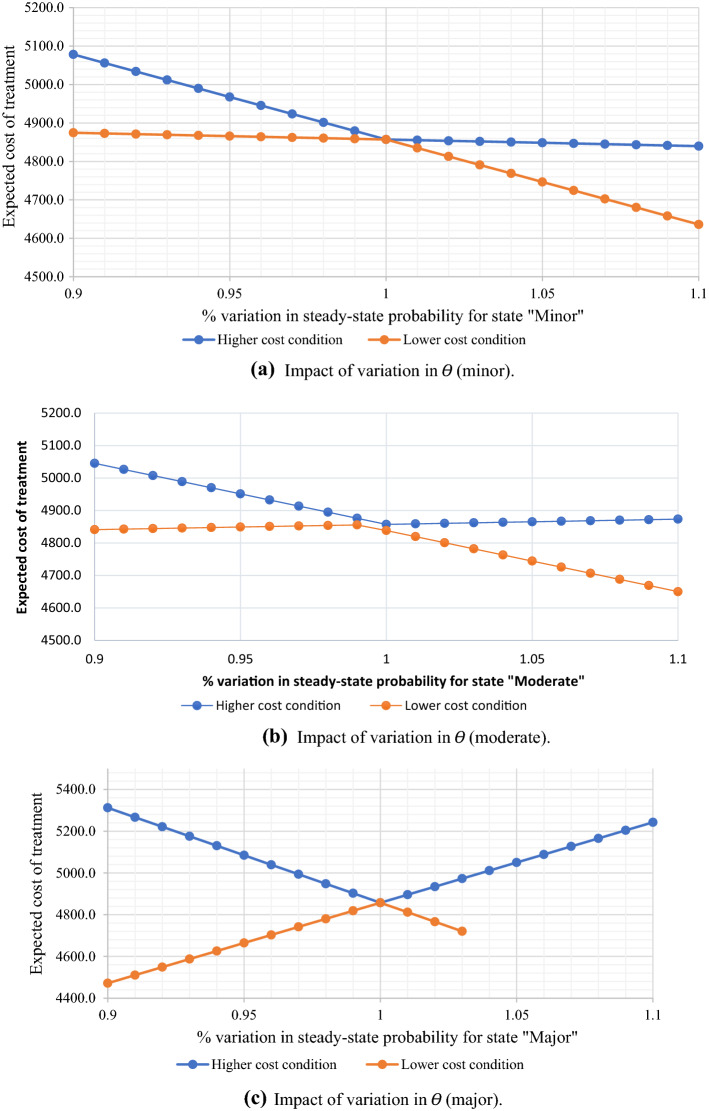

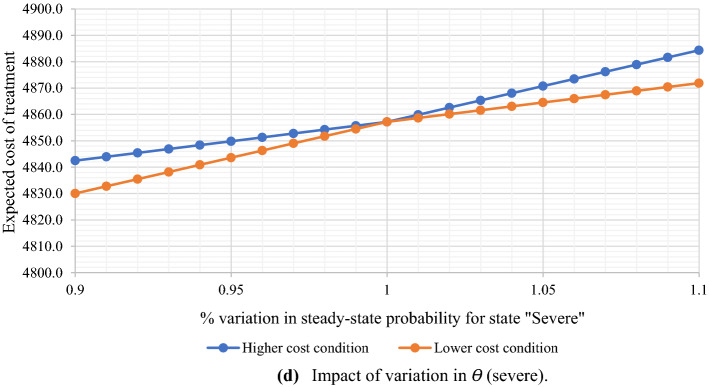
Impact of variations in steady-state probabilities for each state on total expected cost

Figure 5 captures variations in the steady-state probabilities for the four states and their corresponding impact on the expected cost of treatment. Referring to Figs. 5a–d, it can be seen that under higher-cost conditions, the expected cost of treatment was most sensitive to variations in θ(major). Further, in the lower cost condition, the expected cost of treatment was most sensitive to θ(major). Further, referring to Fig. 5a, under both higher and lower-cost conditions, an increase in *θ*(minor) from 90 to 110% resulted in a decreasing trend in the expected cost of treatment. Referring to Fig. 5d, under both higher and lower-cost conditions, an increase in *θ*(severe) from 90 to 110% resulted in an increasing trend in the expected cost of treatment.

It can also be observed that the expected cost of treatment was most sensitive to variations in the steady-state probability at the “major” stage of a medical condition as opposed to the “severe” stage of a medical condition, though the steady-state probability of the “severe” state was less than that of the “major” state.

## Implications

### Managerial implications

From a managerial implications perspective, our study can aid medical service providers such as private and public hospitals, practitioners, and surgeons in several important ways. First, the study enables such entities to approach the cost modeling of treatment costs in a structured and scientific manner while considering real-life data. The robust statistical validation performed at the qualitative stage (stage 1) ensures that samples taken are subject to reasonable testing and validation. Such an approach can be helpful to medical service providers in that large countries (especially developing countries) are often associated with challenges to data collection in a structured manner. It remains impossible in many cases to collect data at a larger sample level or even as part of the population level. Thus, our study shows a possible way in which data pertaining to disease prevalence at various stages of severity, accompanying transitions with accompanying frequencies, and true/false treatment can be collected and handled, leading to meaningful conclusions. Second, the analytical part of the study (stage 2) enables parametrization of specific medical decisions to be made under both the randomized and generalized policy. This parametrization can aid medical service providers in developing some guiding decision support systems such that, by including the contextual factors such as the medical history of a patient in line with treatment cost rationalization, optimal medical treatment decisions can be reached. In particular, such an approach can be extremely beneficial in those cases wherein the doctors’/clinicians’ discretion, depending on different patients, is lower.

### Policy implications

Our study augments the extant research in several important ways at a policy level. First, in the context of low- and middle-income countries (LMICs) and from a resource planning perspective, there has been increasing interest in understanding the cost of healthcare programs that can deliver the desired healthcare services to patients (Clarke-Deeler et al., 2019; Goodarzian et al., 2021). The cost modeling detailed in our study can be scaled up to cover a large population with adjustments related to geographical nuances, lifestyles, and so on, thus providing important policy inputs to prioritize health interventions, make informed decisions related to budgeting, and possibly also considering opportunities for improved efficiency. Of particular importance from our study in terms of a policy contribution is the formalization of the cost of treatment, which can aid in earmarking city- and state-level healthcare budgets such that governments can prioritize considerations for other pressing challenges such as fluctuations in costs associated with the ongoing COVID-19 pandemic. Second, conventional activity-based costing methods, as argued for and demonstrated in the studies of Javid et al. (2016) and McBain et al. (2016), characterize a bottom-up approach, thus requiring significant efforts in painstakingly capturing the cost drivers in the longer term. Instead, our devised approach takes a macro-level perspective of economic cost modeling and considers transition probabilities, decision costs, and types. Third, from the perspective of service providers (both public and private), the framework developed in the study also aids in understanding the relationship between the expected cost of treatment and transition probabilities. For instance, based on cost modeling and sensitivity analysis, it was determined that transitions to “severe” states significantly impact the expected costs of treatment as opposed to transitions to other states. Therefore, with this understanding, governments and private healthcare service providers can sensitize patients about non-medical interventions such as lifestyle changes such that the transitions to “severe” states remain at a minimum. For less life-threatening medical conditions and from a policy perspective, deployment of the randomized policy can be of particular value in that treatment decisions would predominantly be cost-focused. Finally, as many LMICs move toward universal health coverage, the ability to synthesize and visualize disease transitions and other nuances such as the accuracy of medical treatment with robust cost analytics would enable sound financial planning that can be empirically justified.

## Concluding remarks and future research pathways

In this two-stage research, we developed mathematical frameworks to determine the expected cost of treatment per patient in the long-term, considering the integration of various interrelated nuances such as the transition of the medical condition, accuracy of medical treatment, and medical decisions taken at various severity levels of the medical conditions. Further, LP-based and exact method-oriented modeling approaches were deployed to ascertain the steady-state probabilities corresponding to the respective severity levels of the medical condition under both the deterministic and randomized policy. However, before delving into the modeling aspect of the study, thereby taking into context the prescriptive setting and at the qualitative stage of the study, we also focused on the data collection and validation of various cogent hypotheses, thus providing input to the modeling stage of research. To this end and to ensure a strong empirical underpinning to the research, we relied on the data collected from 115 different cardiovascular medical professionals to understand the nuances related to disease transition, the accuracy of medical treatment, and treatment decisions about individual disease severity levels. In particular, we relied on semi-structured interviews and thematic analysis to understand the characteristics related to the empirical setting. Based on a few key hypotheses developed and their subsequent validation, we utilized the empirical data as an input to the modeling stage of the study. Thematically, there were four broad severity levels of the cardiovascular condition identified: “minor,” “moderate,” “major,” and “severe.”

At both the qualitative and prescriptive modeling stage of the study, several interesting insights emerged based on the case example of a history of cardiovascular treatment at the service facilities of a well-known multi-specialty hospital chain in Eastern India. For instance, at the qualitative stage of the research, it was determined that treatment accuracy was better in more severe states of cardiovascular conditions and inferior in relatively less severe states of the condition. Counter-intuitively, it was also determined that the probabilistic value of the transition from “moderate” to “severe” was higher compared with the transition from “major” to “severe”. At the prescriptive modeling stage, though one of our primary contributions relates to developing the novel mathematical framework, with subsequent optimization runs, we illustrated that the randomized policy seems to be cost-competitive compared with the deterministic policy. Further, using a sensitivity analysis, we showcased the impact of the varying steady-state probabilities of the respective states of a medical condition on the expected cost of treatment. Finally, there are several ways our study can be aligned with favorable policy implications, one of which is possibly considering a randomized treatment policy in LMIC countries to treat pervasive but less life-threatening conditions.

Like any study, ours is also not devoid of limitations. First, our research only considered direct costs when modeling the expected cost of treatment. Other indirect costs, such as wages, administrative costs, and so on, were not considered. This implication is particularly important for countries like the US, wherein administrative costs typically constitute a significant proportion of overall healthcare expenditure. Second, in demonstrating our modeling framework, the sample data used was rather limited and primarily belonged to the same geographical setting and similar age group. Therefore, to further generalize the study's findings, it is imperative that the framework developed to be tested in a larger setting with a more heterogeneous population. Third, at the qualitative stage of our study, we relied on respondents’ inputs to ascertain the transitions pertinent to the medical condition and the accuracy of the treatment. An implicit assumption here is that the data did not significantly suffer from the clinicians’ biases in that the IDEA framework works effectively in such cases. Fourth, another future research direction would be to explore the application of more advanced supervised learning methods such as deep learning and structure analysis to improve the performance of cost prediction methods. Such forecasts can be conducted with respect to certain extant data over a sufficiently long period. Specifically, adding the features of medical treatment and benefiting from their predictive and explanatory power can be an important step in such approaches.

## Figures and Tables

**Table 10:**
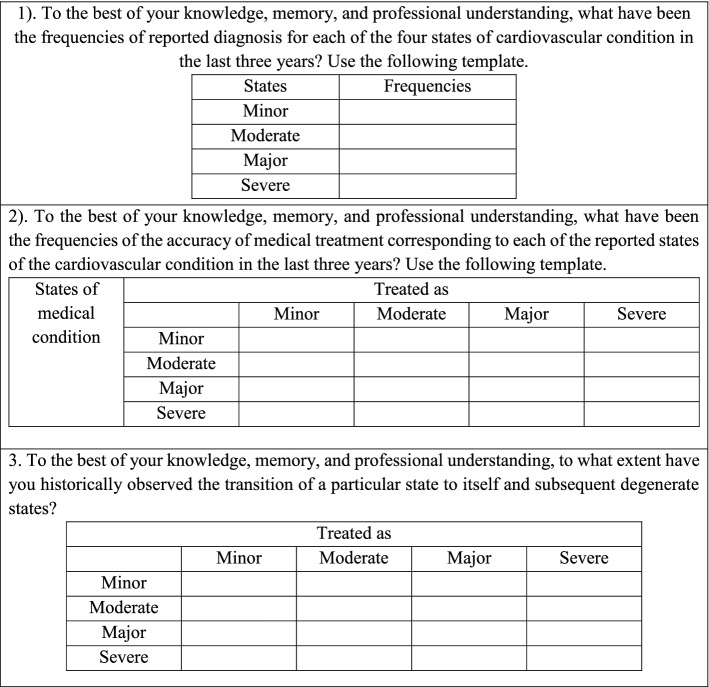
Excerpt of the survey questionnaire

## Appendix

See Tables 9 and 10.

**Table 9:** Study and clinicians’ attributes

Attribute type	Sub-attribute	Detail/value
Related to clinicians	Expert profile	Retired cardio/heart specialist and surgeon
	Minimum professional experience	22 years
	Maximum professional experience	34 years
	Number of clinicians reached out to	187
	Number of clinicians agreed to share inputs	115
	Highest education attainment	MD or DM in cardiology
Related to the study	Video platform used for a semi-structured interview	Cisco Webex
	Minimum duration of the interview	43 min
	Maximum duration of the interview	57 min
	Average duration of the interview	51 min
	Period of the study	July to December 2020

## References

[CR1] Balta M, Valsecchi R, Papadopoulos T, Bourne DJ (2021). Digitalization and co-creation of healthcare value: A case study in Occupational Health. Technological Forecasting and Social Change.

[CR2] Beaulieu M, Bentahar O (2021). Digitalization of the healthcare supply chain: A roadmap to generate benefits and effectively support healthcare delivery. Technological Forecasting and Social Change.

[CR3] Braun V, Clarke V (2006). Using thematic analysis in psychology. Qualitative Research in Psychology.

[CR4] Chapel JM, Ritchey M, Zhang D, Wang G (2017). Prevalence and medical costs of chronic diseases among adult Medicaid beneficiaries. American Journal of Preventative Medicine.

[CR5] Clarke-Deeler E, Vassall A, Menzies NA (2019). Estimators used in multisite healthcare costing studies in low- and middle-income countries: A systematic review and simulation study. Value in Health.

[CR6] Constantinou AC, Fenton N, Marsh W, Radlinski L (2016). From complex questionnaire and interviewing data to intelligent Bayesian network models for medical decision support. Artificial Intelligence in Medicine.

[CR7] Cookson R, Mirelman AJ, Griffin S, Asaria M, Dawkin B, Norheim OF, Verguet SV, Culyer A (2017). Using cost-effectiveness analysis to address health equity concerns. Value in Health.

[CR50] Cooper, L. A., Beach, M. C., Johnson, R. L., & Inui, T. S. (2006). Delving below the surface. *Journal of General Internal Medicine*, *21*(1), 21–27. 10.1111/j.1525-1497.2006.00305.x PMC1484840 16405705

[CR8] Cooper N, Lambert PC, Abrams KR, Sutton AJ (2007). Predicting costs over time using Bayesian Markov chain Monte Carlo methods: An application to early inflammatory polyarthritis. Health Economics.

[CR9] Daultani Y, Chaudhuri A, Kumar S (2015). A decade of lean in healthcare: Current state and future directions. Global Business Review.

[CR10] Daultani Y, Kumar S, Vaidya O (2015). Improving out-patient flow at an Indian ophthalmic hospital. Operations and Supply Chain Management: An International Journal.

[CR11] de Gues SWL, Evans DB, Eskander MF, Smith JK, Wolff RA, Miksad RA, Weinstein MC, Tseng JF (2016). Neoadjuvant therapy versus upfront surgical strategies in resectable pancreatic cancer: A Markov decision analysis. European Journal of Surgical Oncology.

[CR12] Dhagarra D, Goswami M, Sarma PRS, Choudhury A (2019). Big data and blockchain supported conceptual model for enhanced healthcare coverage: The Indian context. Business Process Management Journal.

[CR13] Douglas T (2020). Responsibility-sensitive healthcare funding: Three responses to Clavien and Hurst’s critique. Cambridge Quarterly of Healthcare Ethics.

[CR52] Douglas, M., Katikireddi, S. V., Taulbut, M., McKee, M., & McCartney, G. (2020). Mitigating the wider health effects of covid-19 pandemic response. *BMJ*, *369*, m1557. 10.1136/bmj.m1557 PMC7184317 32341002

[CR14] Goldkuhl G (2012). Pragmatism vs interpretivism in qualitative information systems research. European Journal of Information Systems.

[CR15] Goodarzian F, Ghasemi P, Gunasekaren A, Taleizadeh AA, Abraham A (2021). A sustainable-resilience healthcare network for handling COVID-19 pandemic. Annals of Operations Research.

[CR16] Hemming V, Burgman MA, Hanea AM, McBride MF, Wintle BC (2018). A practical guide to structured expert elicitation using the IDEA protocol. Methods in Ecology and Evolution.

[CR17] Henrique DB, Filho MG, Marodin G, Jabbour ABLDS, Chiappetta Jabbour CJ (2021). A framework to assess sustaining continuous improvement in lean healthcare. International Journal of Production Research.

[CR18] Hillier FS, Lieberman GJ, Nag B, Basu P (2010). Introduction to operations research.

[CR19] Javid M, Hadian M, Ghaderi H, Ghaffari S, Salehi M (2016). Application of the activity-based costing method for unit-cost calculation in a hospital. Global Journal of Health Science.

[CR21] Khalilpourazari S, Doulabi HH, Ciftcioglu AO, Weber GW (2021). Gradient-based grey wolf optimizer with Gaussian walk: Application in modelling and prediction of the COVID-19 pandemic. Expert Systems with Applications.

[CR51] Kong, G., Jiang, L., Yin, X., Wang, T., Xu, D. L., Yang, J. B., & Hu, Y. (2018). Combining principal component analysis and the evidential reasoning approach for healthcare quality assessment. *Annals of Operations Research*, *271*(2), 679–699.

[CR23] Kwon I-WG, Kim S-H, Martin DG (2016). Healthcare supply chain management; strategic areas for quality and financial improvement. Technological Forecasting and Social Change.

[CR24] Li ZP, Wang JJ, Chang AC, Shi J (2021). Capacity reallocation via sinking high-quality resource in a hierarchical healthcare system. Annals of Operations Research.

[CR25] Lin S, Zhang Q, Chen F, Lou L, Chen L, Zhang W (2019). Smooth Bayesian network model for the prediction of future high-cost patients with COPD. International Journal of Medical Informatics.

[CR26] Manrique-Rodriguez S, Sanchez-Galindo AC, Lopez-Herce J, Calleja-Hernandez MA, Martinez-Martinez F, Iglesias-Peinado I, Carrillo-Alvarez A, Sanjurjo-Saez M, Fernandez-Llamazares CM (2014). Implementing smart pump technology in a pediatric intensive care unit: A cost-effective approach. International Journal of Medical Informatics.

[CR27] McBain RK, Jerome G, Warsh J, Browning M, Mistry B, Faure PIF, Pierre C, Fang AP, Mugunga JC, Rhatigan J, Leandre F, Kaplan R (2016). Rethinking the cost of healthcare in low-resource settings: The value of time-driven activity-based costing. BMJ Journal of Global Health.

[CR28] Mitropoulos P, Zervopoulos PD, Mitropoulos I (2020). Measuring performance in the presence of noisy data with targeted desirable levels: Evidence from healthcare units. Annals of Operations Research.

[CR29] Morid, M. A., Kawamoto, K., Ault, T., Dorius, J., & Abdelrahman, S. (2018). Supervised learning methods for predicting healthcare costs: Systematic literature review and empirical evaluation. In *AMIA annual symposium proceedings*. PMC5977561 29854200

[CR30] Raghupathi W, Raghupathi V (2018). An empirical study of chronic diseases in the United States: A visual analytics approach to public health. International Journal of Environmental Research and Public Health.

[CR31] Revels S, Kumar SAP, Ben-Assuli O (2017). Predicting obesity rate and obesity-related healthcare costs using data analytics. Health Policy and Technology.

[CR32] Sari N, Rotter T, Goodridge D, Harrison L, Kingsman L (2017). An economic analysis of a system wide Lean approach: Cost estimations for the implementation of Lean in the Saskatchewan healthcare system for 2012–2014. BMC Health Services Research.

[CR33] Saunders M, Lewis P, Thornhil A (2019). Research methods for business students.

[CR34] Šimundić A-M (2009). Measures of diagnostic accuracy: Basic definitions. The Journal of International Federation of Clinical Chemistry and Laboratory Medicine.

[CR35] Stadhouders N, Koolman X, Tanke M, Maarse H, Jeurissen P (2016). Policy options to contain healthcare costs: A review and classification. Health Policy.

[CR36] Sushmita, S., Newman, S., Marquardt, J., Ram, P., Prasad, V., De Cock, M., & Teredesai, A. (2015). Population cost prediction on public healthcare datasets. In *Proceedings of the 5th international conference on digital health 2015*, 10.1145/2750511.2750521.

[CR37] Tortorella GL, Fogliatto F, Sunder MV, Veragara AMC, Vassolo R (2021). Assessment and prioritisation of Healthcare 4.0 implementation in hospitals using Quality Function Deployment. International Journal of Production Research.

[CR38] van Baal P, Meltzer D, Brouwer W (2014). Future costs, fixed healthcare budgets, and the decision rules of cost-effectiveness analysis. Health Economics.

[CR39] Verma A, Kuo YH, Kumar MM, Pratap S, Chen V (2022). A data analytic-based logistics modelling framework for E-commerce enterprise. Enterprise Information Systems.

[CR40] Walker RC, Tong A, Howard K, Palmer SC (2019). Patient expectations and experiences of remote monitoring for chronic diseases: Systematic review and thematic synthesis of qualitative studies. International Journal of Medical Informatics.

[CR41] Wammes JJG, van der Wees PJ, Tanke MAC, Westert GP, Jeurissen PPT (2018). Systematic review of high-cost patients’ characteristics and healthcare utilisation. British Medical Journal Open.

[CR42] Wang H, Cui Z, Chen Y, Avidan M, Abdallah AB, Kronzer A (2018). Predicting hospital readmission via cost-sensitive deep learning. IEEE/ACM Transactions on Computational Biology and Bioinformatics.

[CR43] Weaver CG, Clement FM, Campbell NRC, James MT, Klarenbach SW, Hemmelgarn BR, Tonelli M, McBrienfor KA (2015). Healthcare costs attributable to hypertension. Hypertension.

[CR44] Wellington J, Szczerbinski M (2007). Research methods for the social sciences.

[CR45] WHO Working Paper. (2019). *Global spending on health: A world in transition*. World Health Organization. (WHO/HIS/HGF/HFWorkingPaper/19.4). https://apps.who.int/iris/bitstream/handle/10665/330357/WHO-HIS-HGF-HF-WorkingPaper-19.4-eng.pdf. Accessed on October 17, 2021.

[CR46] World Health Organization. (2022a). Health systems and governance: Economic evaluation & analysis. https://www.who.int/teams/health-systems-governance-and-financing/economic-analysis, Accessed April 16, 2022a.

[CR47] World Health Organization. (2022b). Health systems and governance: Costing and technical efficiency. https://www.who.int/teams/health-systems-governance-and-financing/economic-analysis/costing-and-technical-efficiency. Accessed April 16, 2022b.

[CR48] Zhao G, Hormazhabal JH, Elgueta S, Manzur JP, Liu S, Chen H, Lopez C, Karturiratne D, Chen X (2020). The impact of knowledge governance mechanisms on supply chain performance: Empirical evidence from the agri-food industry. Production Planning and Control.

